# A Systematically Reduced Mathematical Model for Organoid Expansion

**DOI:** 10.3389/fbioe.2021.670186

**Published:** 2021-06-10

**Authors:** Meredith A. Ellis, Mohit P. Dalwadi, Marianne J. Ellis, Helen M. Byrne, Sarah L. Waters

**Affiliations:** ^1^Mathematical Institute, University of Oxford, Oxford, United Kingdom; ^2^Department of Chemical Engineering, University of Bath, Bath, United Kingdom; ^3^Cellesce, Cardiff Medicentre, Heath Park, Cardiff, United Kingdom

**Keywords:** organoid culture, bioreactor, asymptotic, multiscale, transport, reduced-order model

## Abstract

Organoids are three-dimensional multicellular tissue constructs. When cultured *in vitro*, they recapitulate the structure, heterogeneity, and function of their *in vivo* counterparts. As awareness of the multiple uses of organoids has grown, *e.g*. in drug discovery and personalised medicine, demand has increased for low-cost and efficient methods of producing them in a reproducible manner and at scale. Here we focus on a bioreactor technology for organoid production, which exploits fluid flow to enhance mass transport to and from the organoids. To ensure large numbers of organoids can be grown within the bioreactor in a reproducible manner, nutrient delivery to, and waste product removal from, the organoids must be carefully controlled. We develop a continuum mathematical model to investigate how mass transport within the bioreactor depends on the inlet flow rate and cell seeding density, focusing on the transport of two key metabolites: glucose and lactate. We exploit the thin geometry of the bioreactor to systematically simplify our model. This significantly reduces the computational cost of generating model solutions, and provides insight into the dominant mass transport mechanisms. We test the validity of the reduced models by comparison with simulations of the full model. We then exploit our reduced mathematical model to determine, for a given inlet flow rate and cell seeding density, the evolution of the spatial metabolite distributions throughout the bioreactor. To assess the bioreactor transport characteristics, we introduce metrics quantifying glucose conversion (the ratio between the total amounts of consumed and supplied glucose), the maximum lactate concentration, the proportion of the bioreactor with intolerable lactate concentrations, and the time when intolerable lactate concentrations are first experienced within the bioreactor. We determine the dependence of these metrics on organoid-line characteristics such as proliferation rate and rate of glucose consumption per cell. Finally, for a given organoid line, we determine how the distribution of metabolites and the associated metrics depend on the inlet flow rate. Insights from this study can be used to inform bioreactor operating conditions, ultimately improving the quality and number of bioreactor-expanded organoids.

## 1. Introduction

Organoid technology is becoming increasingly prominent as a biomedical tool, with applications in drug discovery and personalised medicine. In biomedical research, brain, kidney, and liver organoids are used to understand the underlying biological mechanisms in tissue development and tissue-drug interactions (Eisenstein, 2018; Kondo and Inoue, 2019; Tuveson and Clevers, 2019; Bock et al., 2020).

Organoids are three-dimensional, multicellular structures which, when grown *in vitro*, recapitulate the structure, function, and heterogeneous cellular composition of *in vivo* tissues (Drost and Clevers, 2018). Their three-dimensional geometry means they are more representative of *in vivo* tissues than 2D cell cultures (Young and Reed, 2016). “Organoid expansion” refers to the growth of multiple organoids from pluripotent stem cells, which are typically derived from patient biopsies or from other organoids (de Souza, 2018). The stem cells are embedded in a supporting extra-cellular matrix (ECM) and cultured in carefully-controlled conditions designed to promote organoid growth. The surrounding ECM provides the biochemical and biomechanical cues needed for the cells to proliferate and differentiate into specialised cells, as happens *in vivo* (Huang et al., 2012; Eisenstein, 2018).

Current methods for organoid expansion are labour intensive, with organoids typically being produced in small numbers at specialist research laboratories. New technologies are required to manufacture large numbers of organoids with uniform and reproducible characteristics, to meet the demands of applications such as high-throughput screening in drug development. One such technology exploits bioreactors, which aim to deliver sufficient nutrients and growth factors to the cells to promote cell proliferation and differentiation, and to prevent the accumulation of toxins, which can lead to cell death. For a more detailed overview of bioreactor technologies used for 3D cell culture see, for example, Martin et al. (2004), Pörtner and Giese (2006), and Wendt et al. (2009).

**Figure 1:**
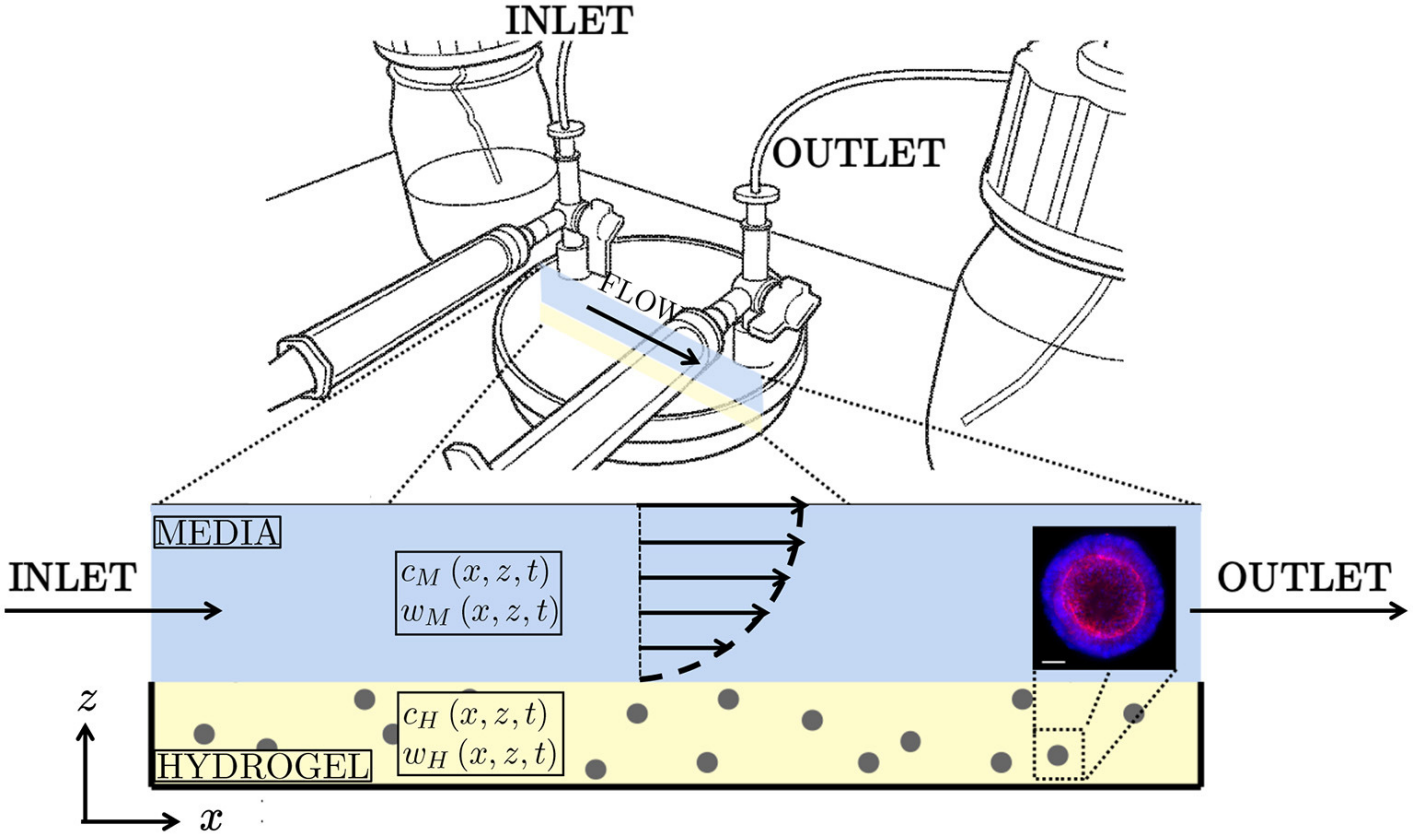
**(Top)** Schematic of “CXP1” bioreactor (Ellis et al., 2019). **(Bottom)** Two-dimensional cross-section of the bioreactor, with arrows indicating the half-Poiseuille flow profile. Blue is media, yellow is hydrogel, grey is organoid biomass. The glucose concentrations within the media and hydrogel are given by *c*_*M*_ and *c*_*H*_, respectively. Similarly, the lactate concentrations within hydrogel and media are denoted *w*_*M*_ and *w*_*H*_, respectively. **(Bottom right)** Example of colorectal cancer organoid. Confocal image using 20X objective of Cell Insight Cx7. Organoid stained for nuclear (blue) and cytoskeletal (red) markers for imaging. Scalebar 50 μm. Reproduced with permission from Cellesce.

This study is motivated by proprietary organoid expansion bioreactor technology developed by Cellesce (Ellis et al., 2019). The “Cellesce Expansion 1 (CXP1)” bioreactor is currently used to expand colorectal cancer organoids, see Figure 1. Flow of media through the system enhances the delivery of nutrients to, and the removal of waste products from, organoids seeded in a hydrogel layer. In this application, oxygen is present at high concentrations, and is not a limiting factor for organoid growth. The key metabolites of interest here are glucose, essential for colorectal cancer organoid growth, and lactate. Lactate can have a detrimental effect on cell behaviour, such as metabolism (Romero-Garcia et al., 2016), and sufficiently high levels can lead to cell death. Lactate can be produced via anaerobic respiration and aerobic glycolysis (Liberti and Locasale, 2016). We do not focus on the precise mechanisms of lactate production here, but instead determine how the media flow promotes lactate removal. We note that while colorectal cancer organoids tolerate high lactate concentrations, the intention is to use CXP1 to expand a range of normal (healthy) and pathological organoids. Since different organoid types have distinct requirements (*e.g*. nutrient levels required for cell proliferation and lactate tolerances), understanding the mass transport of glucose and lactate within the bioreactor is important. While we acknowledge the biological complexity of organoid culture, spatiotemporal knowledge of these two metabolites provide useful and practical information on the operation of CXP1, and provides the framework for more complex models in the future.

Key priorities in the CXP1 bioreactor design and operation are uniformity of organoid size and system reproducibility, to ensure there is minimal variation in organoid characteristics between and within batches grown under the same operating conditions. The main control parameters for the CXP1 bioreactor are the inlet flow rate (controlled via a peristaltic pump) and the initial cell seeding density (the organoids are grown from single cells). Optimisation of these control parameters requires spatiotemporal information about the flow and metabolite (here glucose and lactate) concentrations throughout the bioreactor (Galban and Locke, 1999a). Such data are impractical, inefficient, and expensive to collect through experimental means alone.

To complement experimental studies, mathematical models of bioreactor systems can be used to predict media flow profiles and the associated metabolite concentrations that cannot easily be measured *in vitro*, thus providing useful insights to ensure CXP1 operation is maintained within tolerable operating regions of these metabolites. Here, we adopt a continuum modelling approach, in which the dependent variables (cell density, fluid velocity, metabolite concentrations) are assumed to vary continuously in space and time. Our resulting model comprises a system of partial differential equations (PDEs). A key advantage of such a mathematical modelling approach is the ability to quickly, efficiently and accurately analyse the system as control parameters are varied. A continuum, rather than discrete, cell-based approach is often used to model bioreactor systems, which is justified due to the typical cell numbers ($O\left(10^{6}\right)$ cells) and metabolite concentrations (CXP1: 16 mM in 15 mL of culture media) present. We model the organoids (cell aggregates) as effective (bulk) reaction terms over the hydrogel, which can be formally obtained through an asymptotic homogenisation procedure (see, for example, Dalwadi et al., 2018; Dalwadi and King, 2020).

Here we review existing mathematical models for metabolite transport in bioreactor systems. A variety of different mathematical modelling approaches have been applied to related problems in tissue engineering, including: ordinary differential equation (ODE) models (Sachs et al., 2001); PDE models (Galban and Locke, 1999a,b; Shipley et al., 2009, 2011; Shipley and Waters, 2012; Chapman et al., 2014, 2017; Pearson et al., 2014); computational approaches (Nguyen et al., 2018; Mehrian et al., 2020b); and agent-based models (Drasdo and Höhme, 2005; Byrne et al., 2007; Byrne and Drasdo, 2009). For a more comprehensive review of continuum modelling approaches for tissue engineering, see O'Dea et al. (2012). As noted above, in this work we use a continuum modelling approach to develop a PDE model for metabolite transport within a specific bioreactor set-up. We focus on a systematic model reduction of this model, taking an approach similar to that used in Shipley et al. (2011), Shipley and Waters (2012), and Chapman et al. (2017). In so doing, we highlight two key advantages of model reduction. First, we identify the physical mechanisms that dominate the system behaviour on the timescale of interest. Secondly, reduced models are more tractable than their full model counterpart and, as such, can be solved more rapidly numerically or, in some cases, analytically. This facilitates more detailed exploration of parameter space, which is important for subsequent optimisation of bioreactor operating conditions, and allows more detailed biological models to be incorporated.

We develop a mathematical model of the CXP1 system, with the goal of determining how glucose and lactate levels within the CXP1 bioreactor change as the operating conditions (*e.g*. media inlet flow rate and cell seeding density), and organoid growth characteristics, vary. We introduce a reaction-advection-diffusion system for glucose and lactate transport in the CXP1 bioreactor. The hydrogel and media are viewed as two distinct regions, coupled by interfacial conditions. We restrict attention to a two-dimensional slice through the bioreactor, and obtain numerical solutions to the governing equations. Motivated by typical parameter values of the bioreactor, we perform an asymptotic analysis to systematically reduce the model from a two-dimensional geometry to a one-dimensional model, in which vertically-averaged concentration profiles vary with horizontal position along the length of the bioreactor. We validate this reduced model through successful comparisons with numerical solutions of the full system. We exploit the reduced models to explore the parameter space of cell characteristics and bioreactor operating regimes. To assess glucose and lactate levels, we introduce the following quantitative, time-dependent metrics: *glucose conversion* (the ratio between the total amounts of consumed and supplied glucose); *maximum lactate concentration* within the bioreactor; *proportion of domain with intolerable lactate levels* (*i.e*. lactate levels above a tolerated concentration); and *time when intolerable lactate levels are first experienced*. For a given organoid type, we determine how these metrics change as the inlet flow rate varies. In this way, we aim to show how quantitative insights gained from this modelling approach can inform the selection of experimental bioreactor operating conditions, and ultimately improve the quality and quantity of bioreactor-expanded organoids.

The structure of the paper is as follows. In the Methods section, we introduce the full mathematical model, and then systematically derive two reduced models (referred to as the *longwave approximation* and the *sublimit approximation*) for glucose and lactate transport within the bioreactor. In the Results section, we verify that simulations of the reduced models are in good agreement with solutions of the full model for physiologically relevant parameter regimes. We demonstrate the advantages of the model reductions, highlighting, in particular, the physical insights obtained from systematic derivation of the reduced models from the full system. We then use the longwave approximation model to investigate how the glucose and lactate concentrations within the bioreactor change for different organoid lines. We examine the evolution of the concentration profiles and demonstrate how our quantitative metrics to assess metabolite behaviour are heavily dependent on organoid line characteristics, such as proliferation and nutrient consumption rates. We then investigate, for a specific organoid line, how the media inlet flow rate affects the metabolite concentrations, and explain how this information can be used to optimise the bioreactor control parameters. The paper concludes with a Discussion where we summarise our results and outline future directions for our modelling approach.

## 2. Methods

We derive an unsteady two-dimensional model for glucose and lactate transport within the CXP1 bioreactor. Schematics of the CXP1 bioreactor and our model geometry are presented in Figure 1. We use COMSOL Multiphysics® to solve the full mathematical model numerically and use the insights provided by the numerical simulations to motivate systematic reductions of the full model. The resulting reduced models are solved using a combination of analytical (method of characteristics) and numerical (Chebfun toolbox and ode45 in MATLAB) techniques.

### 2.1. Bioreactor Set-Up

We consider organoids grown from single cells seeded in a homogeneous thin layer of hydrogel in the bioreactor (lower yellow layer in Figure 1). A typical initial seeding density for the CXP1 bioreactor is 4 × 10^5^ cell mL^−1^-6 × 10^5^ cell mL^−1^. We assume that all cells seeded within the hydrogel are viable and become organoids, and that there is negligible settling (which is a fair assumption given the relative time of the gelation of the well-mixed solution, compared to the settling time of the cells). The hydrogel acts as a porous scaffold for the seeded cells, providing the anchorage for cells and the biomechanical and biochemical cues required for cell growth (Huang et al., 2012). The bioreactor is placed within an incubator which maintains constant temperature, O_2_ (atmospheric levels), and CO_2_ concentration. Nutrient-rich culture media, with typical glucose concentration of 16 mM, is stored in an upstream reservoir and is fed into the system through an inlet pipe, and slowly flows across the bioreactor (upper blue layer in Figure 1), with typical flow velocity of 10^−6^m s^−1^. The media is then removed from the bioreactor through an outlet pipe. The top of the culture media layer is a free surface. We assume there is no flow within the hydrogel. We consider colorectal cancer organoids, which are expanded in the bioreactor for 7 days. The organoids are grown from single stem cells (roughly 10μm in diameter) until they are approximately 40-80 μm in diameter and comprise approximately 50 cells. The organoids are then extracted from the hydrogel and tested for size, viability, and number of cells per organoid. The total number of organoids per bioreactor is also recorded. Finally, the extracted organoids are frozen and stored for future use (for example, drug assays).

We consider the bioreactor design, *e.g*. the hydrogel and media depths, to be fixed (though modelling can provide insights into the role of system geometry on the resulting metabolite concentrations). The glucose concentration in the upstream reservoir is also fixed. The bioreactor operating parameters that can be varied are the media inlet flow rate and the cell seeding density in the hydrogel. The key biological question we seek to answer using mathematical modelling is “how do the bioreactor operating conditions and cell characteristics influence the glucose and lactate concentrations within the CXP1 bioreactor.”

#### 2.1.1. Parameter Values

The CXP1 geometry and relevant parameter values (*e.g*. bioreactor length, hydrogel and culture media layer depths, maximum culture media flow velocity, and initial cell seeding density) are outlined in Ellis et al. (2019) and stated in Table 1. The hydrogel used in the CXP1 protocol is Corning Matrigel Matrix and the culture media is a modified form of Dulbecco's modified Eagle medium (DMEM), both of which are described in Ellis et al. (2019).

**Table 1:** Definitions of dimensional model parameters, together with typical values.

**Parameter**	**Definition**	**Typical value**
*D*_*CH*_	Diffusivity of glucose in hydrogel	6.0 × 10^−10^m^2^ s^−1^ (Suhaimi et al., 2015)
*D*_*CM*_	Diffusivity of glucose in media	6.0 × 10^−10^m^2^ s^−1^ (Suhaimi and Das, 2016)
*D*_*WH*_	Diffusivity of lactate in hydrogel	1.2 × 10^−9^m^2^ s^−1^ (Zhou et al., 2008)
*D*_*WM*_	Diffusivity of lactate in media	1.4 × 10^−9^m^2^ s^−1^ (Shipley et al., 2011)
*c*_−∞_	Glucose concentration in upstream reservoir	0.36 mol m^−2^
[*u*]	Maximum velocity of media flow	1 × 10^−6^ m s^−1^
*L*	Length of bioreactor	9 × 10^−2^m
*h*_*H*_	Height of hydrogel layer	1 × 10^−3^m
*h*_*M*_	Combined height of hydrogel and media	3 × 10^−3^m
*N*_0_	Initial cell seeding density	2.7 × 10^10^cell m^−2^ to 4 × 10^10^cell m^−2^
*p*	Proliferation rate	3.9 × 10^−6^s^−1^
ν_*C*_	Rate of glucose consumption per unit cell density	9.4 × 10^−17^m^2^ cell^−1^ s^−1^

*Where no citation is given, parameters are taken from the CPX1 set-up*.

The diffusivities of glucose and lactate in hydrogel and media used in our model are taken from the literature (see Table 1). Our model can be specialised for different cell lines, via characterisation of their rates of proliferation and glucose consumption. In Table 1, we state typical values for rates of cell proliferation and glucose consumption, estimated from CXP1 experimental data of several colorectal cancer organoid cell lines. We were also able to obtain averaged values for lactate concentration in the culture media layer at the end of the experiment empirically, which are similar to the values predicted by the model. Estimating model parameter values from experimental data can be challenging, although there have been advances in predicting cellular proliferation rates, *e.g*. via machine learning methods (Mehrian et al., 2020a).

While the current CXP1 operating conditions have been empirically chosen to be specialised for colorectal cancer organoids, a key advantage of mathematical modelling is that it facilitates consideration of metabolite transport within CXP1 for other cell lines (which is the intent of Cellesce). This knowledge will streamline the adaptation of the CXP1 bioreactor to expanding organoids with significantly different behaviour, *e.g*. non-cancerous organoids.

### 2.2. Mathematical Model

#### 2.2.1. Governing Equations

Motivated by the specific bioreactor set-up, parameter values, cell densities, and metabolite concentrations, discussed in section 2.1, we neglect stochastic effects and adopt a continuum modelling approach. We consider a two-dimensional slice of the bioreactor, and adopt a Cartesian coordinate system ***x*** = (*x, z*) with origin at the bottom-left corner of the domain (see Figure 1). We denote time by *t*. The hydrogel region of the bioreactor is (*x, z*) ∈ [0, *L*] × [0, *h*_*H*_] (yellow region in Figure 1) and the media region is (*x, z*) ∈ [0, *L*] × [*h*_*H*_, *h*_*M*_] (blue region in Figure 1). We denote the glucose concentration by *c* = *c*(*x, z, t*) and the lactate concentration by *w* = *w*(*x, z, t*), with subscripts *M* and *H* to denote concentrations in the media and hydrogel, respectively. We define the model parameters introduced below, together with their typical values, in Table 1.

In the hydrogel, the glucose and lactate are transported via diffusion and glucose is consumed by organoids, which subsequently produce lactate. For the organoids (cell aggregates), we model the reaction terms through effective (bulk) sink/source terms over the hydrogel. Such an approach can be mathematically justified through a formal averaging procedure, such as the asymptotic homogenisation carried out for related systems in Dalwadi et al. (2018) and Dalwadi and King (2020). The equations governing metabolite transport within the hydrogel, (*x, z*) ∈ [0, *L*] × [0, *h*_*H*_], are then:

(2.1) $$\begin{array}{l} \frac{\partial c_{H}}{\partial t}=D_{CH}\nabla^{2}c_{H}-r\left(t,x,c_{H},w_{H}\right)n\left(t\right), \end{array}$$

(2.2) $$\begin{array}{l} \frac{\partial w_{H}}{\partial t}=D_{WH}\nabla^{2}w_{H}+s\left(t,x,c_{H},w_{H}\right)n\left(t\right), \end{array}$$

where *r* and *s* denote the rates of glucose consumption and lactate production per cell, respectively (units mol cell^−1^ s^−1^) and *n*(*t*) is the cell density at time *t* (units cell m^−2^). We assume the cells proliferate at rate *p*, so that the cell density is

(2.3) $$\begin{array}{l} n\left(t\right)=N_{0}e^{pt}, \end{array}$$

where *N*_0_ is the spatially uniform initial cell-seeding density. While cell growth is likely to have some dependence on the glucose consumption and local lactate concentration, we assume, as a first approximation, that glucose and lactate concentrations are not growth-rate limiting. Thus, due to the spatially uniform initial cell density, the cell density does not vary in space.

During glycolysis, one glucose molecule produces energy and two lactate molecules (Liberti and Locasale, 2016). Motivated by this, we impose

(2.4) $$\begin{array}{l} s=2r. \end{array}$$

In general, we expect the glucose consumption to be a monotonically increasing function of glucose concentration. For simplicity, we assume that

(2.5) $$\begin{array}{l} r=\nu_{C}c_{H}, \end{array}$$

where ν_*C*_ is a constant (units m^2^ cell^−1^ s^−1^) representing the rate of glucose consumption per unit cell density.

In the media, (*x, z*) ∈ [0, *L*] × [*h*_*H*_, *h*_*M*_], the advection-diffusion equations for metabolite transport are:

(2.6) $$\begin{array}{l} \frac{\partial c_{M}}{\partial t}+u\left(z\right)\frac{\partial c_{M}}{\partial x}=D_{CM}\nabla^{2}c_{M}, \end{array}$$

(2.7) $$\begin{array}{l} \frac{\partial w_{M}}{\partial t}+u\left(z\right)\frac{\partial w_{M}}{\partial x}=D_{WM}\nabla^{2}w_{M}, \end{array}$$

where *u*(*z*) is the horizontal media flow. Given the slow nature of the flow and geometry of the flow domain, the flow is well-approximated by pressure-driven lubrication flow with a free surface, so that *u*(*z*) is the half-Poiseuille flow:

(2.8) $$\begin{array}{l} u\left(z\right)=\left[u\right]\frac{{\left(z-h_{H}\right)}^{2}}{{\left(h_{M}-h_{H}\right)}^{2}}, \end{array}$$

where [*u*] is the maximum flow velocity.

Governing equations Equations (2.1)-(2.8) require appropriate boundary, initial, and interfacial conditions. The boundaries in the hydrogel are solid walls and we impose zero flux of glucose and lactate at *x* = 0, *L*:

(2.9) $$\begin{array}{l} -D_{CH}\frac{\partial c_{H}}{\partial x}=-D_{WH}\frac{\partial w_{H}}{\partial x}=0. \end{array}$$

We assume the concentrations of glucose and lactate in the inlet pipe are maintained at the constant values *c*_−∞_ and 0, respectively. We assume pointwise continuity of metabolite flux at the inlet, *x* = 0:

(2.10) $$\begin{array}{ll} u\left(z\right)c_{M}-D_{CM}\frac{\partial c_{M}}{\partial x}=u\left(z\right)c_{-\infty }, & u\left(z\right)w_{M}-D_{WM}\frac{\partial w_{M}}{\partial x}=0; \end{array}$$

and we impose no diffusive flux of metabolites at the outlet, *x* = *L*:

(2.11) $$\begin{array}{l} -D_{CM}\frac{\partial c_{M}}{\partial x}=-D_{WM}\frac{\partial w_{M}}{\partial x}=0, \end{array}$$

noting that the metabolites can leave the bioreactor via advection. We impose no-flux conditions for the metabolites at the base of the hydrogel, *z* = 0, and at the top of the media layer, *z* = *h*_*M*_:

(2.12) $$\begin{array}{l} -D_{CH}\frac{\partial c_{H}}{\partial z}=-D_{WH}\frac{\partial w_{H}}{\partial z}=0\text{ at }z=0,\\ -D_{CM}\frac{\partial c_{M}}{\partial z}=-D_{WM}\frac{\partial w_{M}}{\partial z}=0\text{ at }z=h_{M}. \end{array}$$

At the media-hydrogel interface, *z* = *h*_*H*_, we impose continuity of metabolite concentration and flux:

(2.13) $$\begin{array}{l} \;c_{M}=c_{H},w_{M}=w_{H},\\ D_{CM}\frac{\partial c_{M}}{\partial z}=D_{CH}\frac{\partial c_{H}}{\partial z},D_{WM}\frac{\partial w_{M}}{\partial z}=D_{WH}\frac{\partial w_{H}}{\partial z}. \end{array}$$

**Figure 2:**
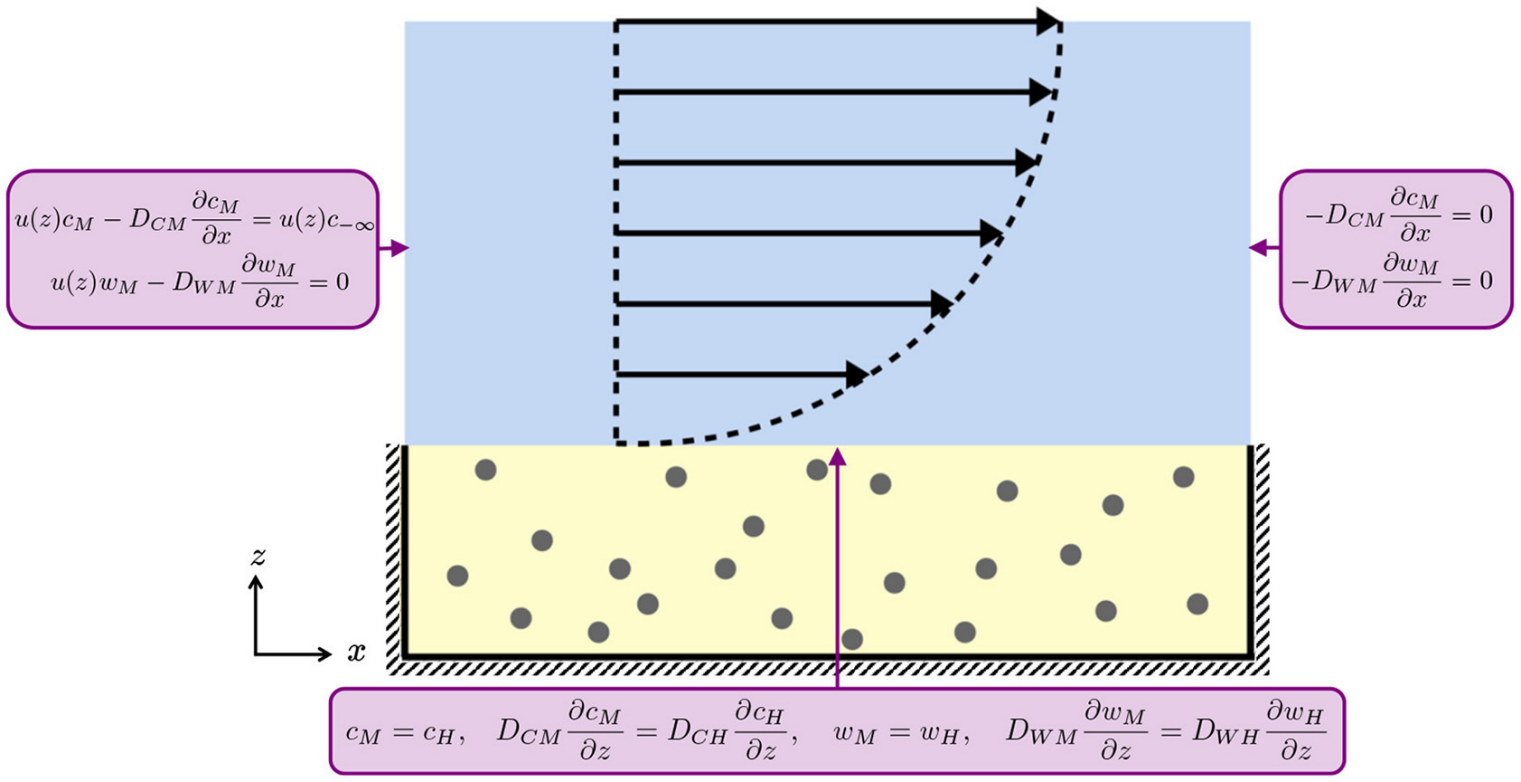
Schematic of the boundary conditions for the media (blue) and hydrogel (yellow) layers for Equations (2.1), (2.2), (2.6), and (2.7). At the media-hydrogel interface, we impose continuity of concentration and flux. At the air-media interface and at the impermeable hashed boundaries, we impose no flux. The black arrows indicate the half-Poiseuille flow profile.

A schematic of these boundary conditions on the domain geometry is given in Figure 2.

As initial conditions, we assume that the glucose concentration in the media equals the glucose concentration in the upstream reservoir, *c* = *c*_−∞_, the glucose concentration in the hydrogel is zero, and that there is no lactate throughout the bioreactor:

(2.14) $$\begin{array}{l} c_{H}=0,\;c_{M}=c_{-\infty },\;w_{H}=w_{M}=0\text{ at }t=0. \end{array}$$

#### 2.2.2. Typical Timescales

The typical parameter values, given in Table 1, reveal that the physical processes included in our model act over three different timescales: hours, days, and months, as shown in Table 2. Diffusion in the *z*-direction occurs over the timescale of hours; media flow, glucose consumption, lactate production, and cell proliferation occur over the timescale of a day; and *x*-diffusion occurs over the timescale of months. This scaling analysis reveals that flow markedly enhances metabolite transport in the *x*-direction and that, within the media, advection dominates diffusive transport of metabolites in the horizontal direction. The separation of timescales renders the system stiff and, as such, care is needed when implementing numerical methods for its solution. At the same time, it leads naturally to the identification of large and small dimensionless parameters which can be exploited for model reduction (see section 2.3).

**Table 2:** Timescale groupings of the various physical processes present in the CXP1 bioreactor.

	**Physical process**	**Timescale**
O(hour)	*z* diffusion glucose in hydrogel	$\frac{\epsilon^{2}L^{2}}{D_{CH}}=1.5\times 10^{4}\text{s}=4.2\text{h}$
	*z* diffusion glucose in media	$\frac{\epsilon^{2}L^{2}}{D_{CM}}=1.5\times 10^{4}$ s=4.2 h
	*z* diffusion lactate in hydrogel	$\frac{\epsilon^{2}L^{2}}{D_{WH}}=7,500$ s = 2.1 h
	*z* diffusion lactate in media	$\frac{\epsilon^{2}L^{2}}{D_{WM}}=6,400$ s = 1.8 h
O(day)	Flow	$\frac{L}{\left[u\right]}=9\times 10^{4}$ s = 25 h
	Glucose consumption	$\frac{1}{\nu_{C}N_{0}}=2.7\times 10^{5}-4\times 10^{5}$ s = 74−110 h
	Lactate production	$\frac{1}{2\nu_{C}N_{0}}=1.3\times 10^{5}-2.0\times 10^{5}$ s = 37−55 h
	Cell proliferation	$\frac{1}{p}=2.6\times 10^{5}$ s = 72 h
O(month)	*x* diffusion glucose in hydrogel	$\frac{L^{2}}{D_{CH}}=1.4\times 10^{7}\text{s}=3,800\text{h}$
	*x* diffusion glucose in media	$\frac{L^{2}}{D_{CM}}=1.4\times 10^{7}$ s = 3,800 h
	*x* diffusion lactate in hydrogel	$\frac{L^{2}}{D_{WH}}=6.8\times 10^{6}$ s = 1,900 h
	*x* diffusion lactate in media	$\frac{L^{2}}{D_{WM}}=5.8\times 10^{6}$ s = 1,600 h

*We use “x” and “z” to denote “vertical” and “horizontal”, respectively. The timescale for each process is the value such that the each dimensionless parameter grouping, defined in Equation (2.21) as the ratio of the timescale of interest to the timescale of the physical process, is equal to one*.

#### 2.2.3. Non-dimensionalisation

We non-dimensionalise the problem to identify the relative importance of each transport mechanism. We introduce the following non-dimensional variables, for *i* ∈ {*H, M*}:

(2.15) $$\begin{array}{l} X=\frac{x}{L},Z=\frac{z}{\epsilon L},T=\frac{t}{\left[t\right]},U\left(Z\right)=\frac{u}{\left[u\right]},\left(C_{i},W_{i}\right)=\frac{\left(c_{i},w_{i}\right)}{c_{-\infty }}, \end{array}$$

where ***X*** = (*X, Z*), ϵ = *h*_*M*_/*L* ≪ 1 is the ratio between vertical and horizontal lengthscales, [*t*] is the timescale, and [*u*] is the maximum flow velocity. The bioreactor domain is then (*X, Z*) ∈ [0, 1] × [0, 1] and the media-hydrogel interface is at dimensionless position *Z* = *H*_*H*_ = :*h*_*H*_/(ϵ*L*). Metabolite concentrations are non-dimensionalised with the upstream reservoir glucose concentration, *c*_−∞_. We fix the timescale of interest to be 1 day, so that we consider the transport on the same timescale as cell growth.

Using the scalings Equation (2.15), the governing equations Equations (2.1)-(2.7) become,

(2.16) $$\begin{array}{l} \epsilon^{2}\frac{\partial C_{H}}{\partial T}=d_{CH}\left(\epsilon^{2}\frac{\partial^{2}C_{H}}{\partial X^{2}}+\frac{\partial^{2}C_{H}}{\partial Z^{2}}\right)-\epsilon^{2}\rho C_{H}e^{PT}, \end{array}$$

(2.17) $$\begin{array}{l} \epsilon^{2}\frac{\partial W_{H}}{\partial T}=d_{WH}\left(\epsilon^{2}\frac{\partial^{2}W_{H}}{\partial X^{2}}+\frac{\partial^{2}W_{H}}{\partial Z^{2}}\right)+2\epsilon^{2}\rho C_{H}e^{PT}, \end{array}$$

for (*X, Z*) ∈ (0, 1) × (0, *H*_*H*_) and

(2.18) $$\begin{array}{l} \epsilon^{2}\frac{\partial C_{M}}{\partial T}+\epsilon^{2}\mu U\left(Z\right)\frac{\partial C_{M}}{\partial X}=d_{CM}\left(\epsilon^{2}\frac{\partial^{2}C_{M}}{\partial X^{2}}+\frac{\partial^{2}C_{M}}{\partial Z^{2}}\right) \end{array}$$

(2.19) $$\begin{array}{l} \epsilon^{2}\frac{\partial W_{M}}{\partial T}+\epsilon^{2}\mu U\left(Z\right)\frac{\partial W_{M}}{\partial X}=d_{WM}\left(\epsilon^{2}\frac{\partial^{2}W_{M}}{\partial X^{2}}+\frac{\partial^{2}W_{M}}{\partial Z^{2}}\right) \end{array}$$

for (*X, Z*) ∈ (0, 1) × (*H*_*H*_, 1), with

(2.20) $$\begin{array}{l} U\left(Z\right)=\frac{{\left(Z-H_{H}\right)}^{2}}{{\left(1-H_{H}\right)}^{2}}. \end{array}$$

The dimensionless parameters in Equations (2.16)-(2.20) are:

(2.21) $$\begin{array}{l} \begin{array}{l} \;\mu =\frac{\left[u\right]\left[t\right]}{L},\;\rho =\left[t\right]\nu_{C}N_{0},\;P=p\left[t\right],\\ \left(d_{CH},d_{CM},d_{WH},d_{WM}\right)=\frac{\left[t\right]}{L^{2}}\left(D_{CH},D_{CM},D_{WH},D_{WM}\right). \end{array} \end{array}$$

We provide a physical interpretation of these dimensionless parameters and their typical values in Table 3. The boundary and initial conditions, Equations (2.9)-(2.14), become:

(2.22) $$\begin{array}{l} -d_{CH}\frac{\partial C_{H}}{\partial X}=0,-d_{WH}\frac{\partial W_{H}}{\partial X}=0\text{ at }X=0,1, \end{array}$$

(2.23a) $$\begin{array}{l} \mu UC_{M}-d_{CM}\frac{\partial C_{M}}{\partial X}=\mu U,\text{ at }X=0 \end{array}$$

(2.23b) $$\begin{array}{l} \mu UW_{M}-d_{WM}\frac{\partial W_{M}}{\partial X}=0\text{ at }X=0, \end{array}$$

(2.24) $$\begin{array}{l} -d_{CM}\frac{\partial C_{M}}{\partial X}=0,-d_{WM}\frac{\partial W_{M}}{\partial X}=0\text{ at }X=1, \end{array}$$

(2.25) $$\begin{array}{l} \frac{\partial C_{H}}{\partial Z}=\frac{\partial W_{H}}{\partial Z}=0\text{ at }Z=0, \end{array}$$

(2.26) $$\begin{array}{l} \frac{\partial C_{M}}{\partial Z}=\frac{\partial W_{M}}{\partial Z}=0\text{ at }Z=1, \end{array}$$

(2.27) $$\begin{array}{l} C_{M}=C_{H},\;W_{M}=W_{H}\text{ at }Z=H_{H}, \end{array}$$

(2.28a) $$\begin{array}{l} d_{CH}\frac{\partial C_{H}}{\partial Z}=d_{CM}\frac{\partial C_{H}}{\partial Z}\text{ at }Z=H_{H}, \end{array}$$

(2.28b) $$\begin{array}{l} d_{WH}\frac{\partial W_{H}}{\partial Z}=d_{WM}\frac{\partial W_{M}}{\partial Z}\text{ at }Z=H_{H}, \end{array}$$

(2.29) $$\begin{array}{l} C_{H}=0,\;C_{M}=1,\;W_{H}=W_{M}=0\text{ at }T=0. \end{array}$$

**Table 3:** Definitions of non-dimensionalised model parameters with their typical values.

**Parameter**	**Definition**	**Typical value**
ϵ	Ratio of vertical to horizontal lengthscales	1/30
*d*_*CH*_	Ratio of timescale of interest to timescale of diffusion of glucose in hydrogel	6.4 × 10^−3^
*d*_*CM*_	Ratio of timescale of interest to timescale of diffusion of glucose in media	6.4 × 10^−3^
*d*_*WH*_	Ratio of timescale of interest to timescale of diffusion of lactate in hydrogel	1.28 × 10^−2^
*d*_*WM*_	Ratio of timescale of interest to timescale of diffusion of lactate in media	1.49 × 10^−2^
μ	Ratio of timescale of interest to timescale of flow	0.96
ρ	Ratio of timescale of interest to that of glucose consumption per cell	0.22-0.32
*P*	Ratio of timescale of interest to timescale of cellular proliferation	1/3
*H*_*H*_	Ratio of hydrogel height to the combined height of hydrogel and media layers	1/3
*W*_tol_	Dimensionless maximum tolerated lactate concentration	0.7

*For the simulations in this paper, we take ρ = 0.27 unless otherwise stated*.

#### 2.2.4. Numerical Solution of Full Model

**Figure 3:**
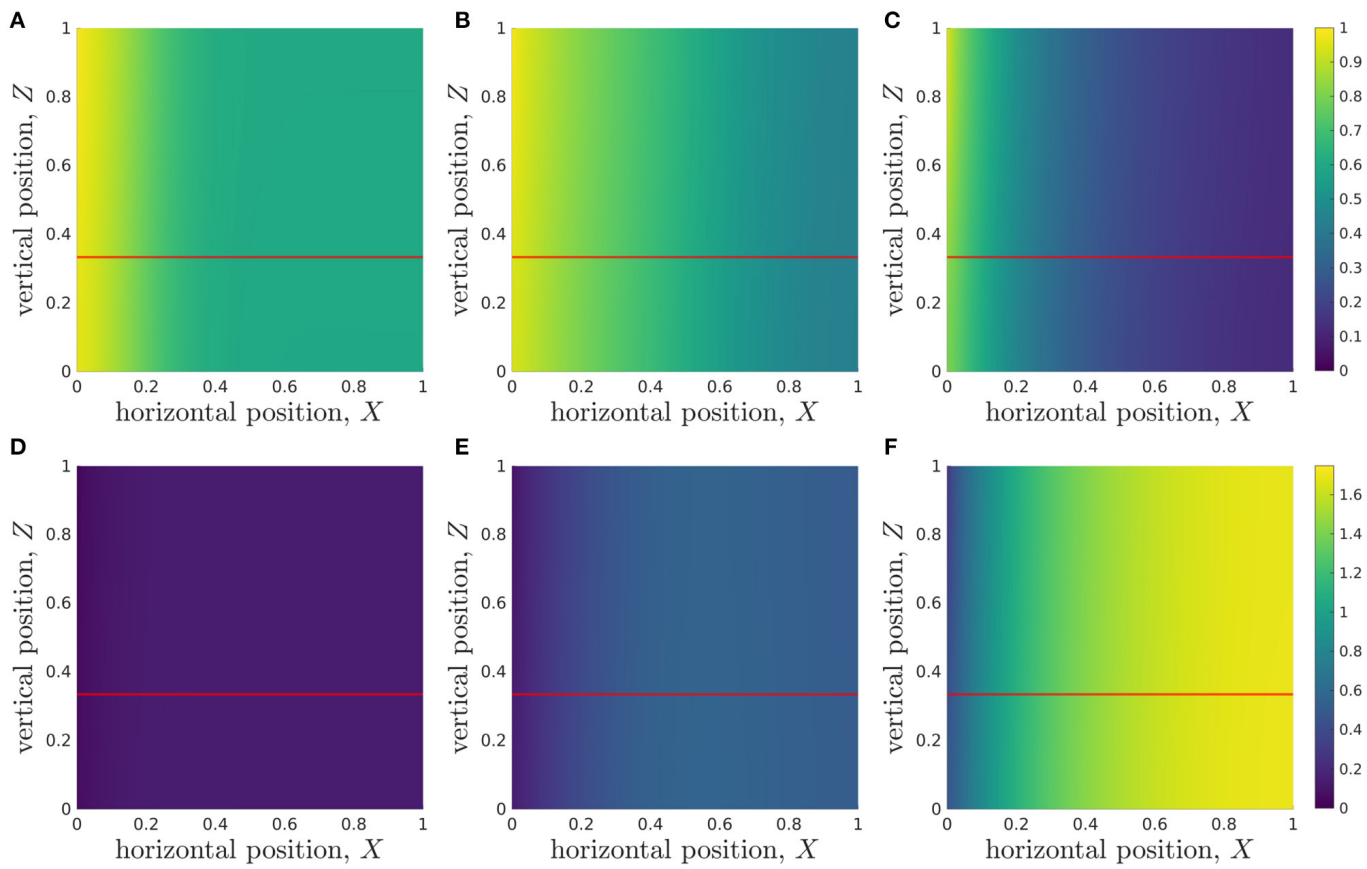
Metabolite concentrations at 1, 3, and 7 days into a typical simulation. The horizontal lines at *Z* = 1/3 represents the media-hydrogel interface. (Top) Glucose distribution *C*(*X, Z, T*) at **(A)** *T* = 1, **(B)** *T* = 3, **(C)** *T* = 7. (Bottom) Lactate distribution *W*(*X, Z, T*) at **(D)** *T* = 1, **(E)** *T* = 3, **(F)** *T* = 7. Parameter values: see Table 3.

We solve the full two-dimensional system, Equations (2.16)-(2.19) and (2.22)-(2.29), using the parameter values given in Table 3, via a finite-element method, using COMSOL Multiphysics® software. The results are checked to be independent of mesh size (results not shown). We plot the metabolite concentration profiles at dimensionless times *T* = 1, 3, 7, corresponding to 1, 3, and 7 days, in Figure 3. Note that we observe little variation in metabolite concentration in the vertical direction for the parameter values given in Table 3.

### 2.3. Model Reduction

As discussed in section 2.2.2, the different transport mechanisms in the system have associated timescales that can be grouped into either hours, days, or months. This is made explicit in the dimensionless system through the presence of the small parameter ϵ. We propose a systematic model reduction, with the key advantage of reducing the complexity of the model while retaining the physical processes which dominate over the timescale of interest.

#### 2.3.1. Longwave Approximation

Motivated by the long, thin geometry of the bioreactor, characterised by ϵ ≪ 1, and the lack of variation in *Z* compared to *X* revealed in Figure 3, we now systematically average Equations (2.16)-(2.19) and (2.22)-(2.29) in *Z* to derive the appropriate reduced lubrication model, referred to as the *longwave approximation*.

In the asymptotic analysis that follows, we consider the limit ϵ → 0, and assume all other dimensionless parameters remain O(1) as ϵ → 0. This distinguished limit is consistent with the values of dimensionless parameters given in Table 3, and assumes that diffusion in the vertical direction is the dominant transport mechanism for the bioreactor geometry. We note that our choice of time scaling, [*t*] = 1 day, means that we are investigating this system over the timescale of days. We could study the behaviour of this system over shorter timescales, and its transition to the timescale of days, if we systematically considered the timescale $T=O\left(\epsilon^{2}\right)$. However, this will not be of fundamental importance to the problem we study here, and we do not pursue this further.

We consider the following asymptotic expansions for the dependent variables:

(2.30) $$\begin{array}{l} f\sim f_{0}+\epsilon^{2}f_{1}+\cdots ,\text{ as }\epsilon \to 0,\text{ where }f\in \left\{C_{M},C_{H},W_{M},W_{H}\right\}. \end{array}$$

We note that the $O\left(\epsilon^{2}\right)$ size of the first-correction term is standard in lubrication-type models, and arises due to the size of the terms neglected in the leading-order problem. In the standard manner, we substitute Equation (2.30) into the governing equations, Equations (2.16)-(2.19) and (2.22)-(2.29), and equate coefficients of $O\left(\epsilon^{n}\right)$.

At leading order, the metabolite transport is given by

(2.31) $$\begin{array}{l} 0=\frac{\partial^{2}f_{j0}}{\partial Z^{2}}\text{ where }f\in \left\{C,W\right\}\text{ and }j\in \left\{H,M\right\}. \end{array}$$

Hence, we see that the leading-order mass transport is driven entirely by vertical diffusion, consistent with our discussion of timescales above.

Integrating Equation (2.31) subject to the leading-order versions of the appropriate boundary conditions, Equations (2.25)-(2.28), we deduce that *C*_*H*0_, *C*_*M*0_, *W*_*H*0_, *W*_*M*0_ are independent of vertical position, *Z*. This is consistent with the numerical solutions seen in Figure 3. Given the continuity of concentration condition, Equation (2.27), we deduce that

(2.32) $$\begin{array}{l} C_{H0}\left(T,X\right)=C_{M0}\left(T,X\right),\;W_{H0}\left(T,X\right)=W_{M0}\left(T,X\right)\text{ for all }Z. \end{array}$$

However, the correct dependence of the metabolite profiles on *T* and *X* is currently undetermined.

To calculate this dependence, we proceed to $O\left(\epsilon^{2}\right)$ and derive an appropriate solvability condition. At $O\left(\epsilon^{2}\right)$, the governing equations are

(2.33) $$\begin{array}{l} d_{CH}\frac{\partial^{2}C_{H1}}{\partial Z^{2}}=\frac{\partial C_{H0}}{\partial T}-d_{CH}\frac{\partial^{2}C_{H0}}{\partial X^{2}}+\rho C_{H0}e^{pT}, \end{array}$$

(2.34) $$\begin{array}{l} d_{WH}\frac{\partial^{2}W_{H1}}{\partial Z^{2}}=\frac{\partial W_{H0}}{\partial T}-d_{WH}\frac{\partial^{2}W_{H0}}{\partial X^{2}}-2\rho C_{H0}e^{pT},\\ \;for\;\left(X,Z\right)\in \left(0,1\right)\times \left(0,H_{H}\right)and \end{array}$$

(2.35) $$\begin{array}{l} d_{CM}\frac{\partial^{2}C_{M1}}{\partial Z^{2}}=\frac{\partial C_{M0}}{\partial T}+\mu U\left(Z\right)\frac{\partial C_{M0}}{\partial X}-d_{CM}\frac{\partial^{2}C_{M0}}{\partial X^{2}}, \end{array}$$

(2.36) $$\begin{array}{l} d_{WM}\frac{\partial^{2}W_{M1}}{\partial Z^{2}}=\frac{\partial W_{M0}}{\partial T}+\mu U\left(Z\right)\frac{\partial W_{M0}}{\partial X}-d_{WM}\frac{\partial^{2}W_{M0}}{\partial X^{2}}\\ \;for\;\left(X,Z\right)\in \left(0,1\right)\times \left(H_{H},1\right). \end{array}$$

Integrating each equation over the vertical coordinate and applying the no flux conditions, Equations (2.25) and (2.26), at $O\left(\epsilon^{2}\right)$ yields:

(2.37) $$\begin{array}{l} d_{CH}\frac{\partial C_{H1}}{\partial Z}{|}_{Z=H_{H}}=H_{H}\left(\frac{\partial C_{H0}}{\partial T}-d_{CH}\frac{\partial^{2}C_{H0}}{\partial X^{2}}+\rho C_{H0}e^{PT}\right), \end{array}$$

(2.38) $$\begin{array}{l} d_{WH}\frac{\partial W_{H1}}{\partial Z}{|}_{Z=H_{H}}=H_{H}\left(\frac{\partial W_{H0}}{\partial T}-d_{WH}\frac{\partial^{2}W_{H0}}{\partial X^{2}}-2\rho C_{H0}e^{PT}\right), \end{array}$$

(2.39) $$\begin{array}{l} -d_{CM}\frac{\partial C_{M1}}{\partial Z}{|}_{Z=H_{H}}=\left(1-H_{H}\right)\left(\frac{\partial C_{M0}}{\partial T}+\mu Ū\frac{\partial C_{M0}}{\partial X}-d_{CM}\frac{\partial^{2}C_{M0}}{\partial X^{2}}\right), \end{array}$$

(2.40) $$\begin{array}{l} -d_{WM}\frac{\partial W_{M1}}{\partial Z}{|}_{Z=H_{H}}=\left(1-H_{H}\right)\left(\frac{\partial W_{M0}}{\partial T}+\mu Ū\frac{\partial W_{M0}}{\partial X}-d_{WM}\frac{\partial^{2}W_{M0}}{\partial X^{2}}\right), \end{array}$$

where the depth-averaged flow velocity, Ū is given by:

(2.41) $$\begin{array}{l} Ū=\frac{1}{1-H_{H}}\int_{H_{H}}^{1}U\left(Z\right)\text{ d}Z=\frac{1}{3}. \end{array}$$

Recalling the continuity of flux condition, Equation (2.28), and that *C*_*H*0_ = *C*_*M*0_ and *W*_*H*0_ = *W*_*M*0_, we combine the above expressions for the glucose and lactate concentrations in the media and hydrogel to derive the *longwave approximation*:

(2.42) $$\begin{array}{l} \alpha \frac{\partial C_{M0}}{\partial T}+\beta \frac{\partial C_{M0}}{\partial X}=\delta_{C}\frac{\partial^{2}C_{M0}}{\partial X^{2}}-\gamma C_{M0}e^{PT}, \end{array}$$

(2.43) $$\begin{array}{l} \alpha \frac{\partial W_{M0}}{\partial T}+\beta \frac{\partial W_{M0}}{\partial X}=\delta_{W}\frac{\partial^{2}W_{M0}}{\partial X^{2}}+2\gamma C_{M0}e^{PT}, \end{array}$$

where we have introduced the following parameters for ease of notation:

(2.44) $$\begin{array}{l} \;\theta =\frac{H_{H}}{1-H_{H}},\alpha =1+\theta ,\beta =\mu Ū,\gamma =\theta \rho ,\\ \delta_{C}=d_{CM}+\theta d_{CH},\delta_{W}=d_{WM}+\theta d_{WH}. \end{array}$$

We derive the appropriate boundary and “initial” conditions for Equations (2.42) and (2.43) in a similar manner, by integrating the leading over terms of Equations (2.22)-(2.24) and (2.29) over *Z* between 0 and 1. We solve Equations (2.42) and (2.43) subject to the following boundary and “initial” conditions:

(2.45) $$\begin{array}{l} \beta C_{M0}-\delta_{C}\frac{\partial C_{M0}}{\partial X}=\beta \text{ at }X=0, \end{array}$$

(2.46) $$\begin{array}{l} \beta W_{M0}-\delta_{W}\frac{\partial W_{M0}}{\partial X}=0\text{ at }X=0, \end{array}$$

(2.47) $$\begin{array}{l} \frac{\partial C_{M0}}{\partial X}=\frac{\partial W_{M0}}{\partial X}=0\text{ at }X=1, \end{array}$$

(2.48) $$\begin{array}{l} C_{M0}=\frac{1}{\alpha }\text{ and }W_{M0}=0\text{ at }T=0\text{ for }0\leq X\leq 1. \end{array}$$

The reason we refer to Equation (2.48) as “initial” conditions is because they actually represent asymptotic matching conditions with the earlier timescale problem we mentioned previously. This is the reason why there is a discontinuity in the boundary and “initial” conditions as *X, T* → 0. If it were of interest to understand this limit further, one could investigate this region using the scalings X=O(ϵ), $T=O\left(\epsilon^{2}\right)$. Given that this asymptotic region does not affect any of our subsequent analysis, for brevity we do not pursue it further here.

Equations (2.42), (2.43), and (2.45)-(2.48) define the longwave approximation model. We will analyse this reduced system in more detail in section 3. First, we derive a further reduction of the longwave approximation, by exploiting the separation in scales between horizontal diffusion and the remaining transport mechanisms, namely advection with the media flow, glucose consumption, and lactate production.

#### 2.3.2. Sublimit of Longwave Approximation

From the typical parameter values given in Table 3, we note that the timescale of horizontal diffusion is significantly longer than the remaining transport mechanisms. Given that the longwave approximation derived in section 2.3.1 is a distinguished asymptotic limit, we can include the separation of scales involved in horizontal diffusion by directly considering the sub-limit *d*_*CH*_, *d*_*CM*_, *d*_*WH*_, *d*_*WM*_ → 0, corresponding to δ_*C*_, δ_*W*_ → 0 in Equations (2.42), (2.43), and (2.45)-(2.48). We refer to this as the *sublimit approximation*. This procedure results in the following governing equations for advection-dominated transport:

(2.49) $$\begin{array}{l} \alpha \frac{\partial C_{M0}}{\partial T}+\beta \frac{\partial C_{M0}}{\partial X}=-\gamma C_{M0}\exp\left(PT\right), \end{array}$$

(2.50) $$\begin{array}{l} \alpha \frac{\partial W_{M0}}{\partial T}+\beta \frac{\partial W_{M0}}{\partial X}=2\gamma C_{M0}\exp\left(PT\right), \end{array}$$

with boundary and initial conditions

(2.51) $$\begin{array}{ll} C_{M0}=1, & \;W_{M0}=0\text{ at }X=0, \end{array}$$

(2.52) $$\begin{array}{ll} C_{M0}=\frac{1}{\alpha }, & \;W_{M0}=0\text{ at }T=0. \end{array}$$

We note that the limit we have taken is singular in that the small parameters (diffusivities) pre-multiply the second-order spatial derivatives. As such, we have lost the ability to prescribe the outlet boundary conditions at *X* = 1, though we note that this boundary condition could be imposed through the analysis of an appropriate (weak) boundary layer near *X* = 1.

A benefit of this sublimit reduction is that we are able to construct analytic solutions for the glucose concentration, using the method of characteristics. The solution is split into two distinct regions: Region 1, given by 0 < β*T* < α*X*; and Region 2, given by 0 < α*X* < β*T*:

(2.53) and (2.54) $$\begin{array}{l} C_{M0}=\{\begin{array}{ll} \frac{1}{\alpha }\exp\left(\frac{\gamma }{\alpha P}\left(1-e^{PT}\right)\right) & \text{ for }0<\beta T<\alpha X,\\ \exp\left(\frac{\gamma }{\alpha P}\left(e^{-P\left(\frac{\alpha }{\beta }X-T\right)}-e^{PT}\right)\right) & \text{ for }0<\alpha X<\beta T, \end{array} \end{array}$$

The solution (2.59)-(2.3.2) is discontinuous across the boundary separating the two regions, *X* = β*T*/α, which we refer to as the *dividing characteristic*. The reason for this is that Region 1 is forced by the initial conditions whereas Region 2 is forced by the boundary conditions, and there is a discontinuity in these conditions near *T* = 0, *X* = 0 (which could be smoothed through an appropriate asymptotic analysis of the earlier timescale, as mentioned previously). As no information from the boundary condition propagates into Region 1, cells in Region 1 do not feel the effect of any replenishment by the flow. As such, we refer to Region 1 as the *unreplenished region* and Region 2 as the *replenished region*.

Using the method of characteristics, we can write the lactate concentration as a single integral of known functions:

(2.55) $$\begin{array}{l} W_{M0}\left(S,\tau \right)=\int_{0}^{\tau }2\gamma C_{M0}\left(T\left(S,\tau \right),X\left(S,\tau \right)\right)e^{PT\left(S,\tau \right)}\text{ d}\tau \\ \text{ with }W_{M0}=0\text{ at }\tau =0, \end{array}$$

where we define the characteristic variables (*S*, τ) as

(2.56) and (2.57) $$\begin{array}{l} S=\alpha X-\beta T\text{ and }\tau =\{\begin{array}{ll} \frac{T}{\alpha } & \text{ for }\beta T<\alpha X,\\ \frac{X}{\beta } & \text{ for }\alpha X<\beta T. \end{array} \end{array}$$

As outlined in the Supplementary Material, we can evaluate the integral in Equation (2.56) to obtain the solution

(2.58) and (2.59) $$\begin{array}{l} W_{M0}=\{\begin{array}{ll} \frac{2}{\alpha }\left(1-\exp\left(\frac{\gamma }{P\alpha }\left(1-e^{PT}\right)\right)\right) & \text{ for }0<\beta T<\alpha X,\\ 2\left(1-\exp\left(\frac{\gamma }{P\alpha }\left(e^{-P\left(\frac{\alpha }{\beta }X-T\right)}-e^{PT}\right)\right)\right) & \text{ for }0<\alpha X<\beta T. \end{array} \end{array}$$

We note that the quantity 2*C*_*M*0_ + *W*_*M*0_ is conserved along the characteristics defined by d*X*/d*T* = α/β (*i.e*. in the advective frame of reference). This means that the following relationships are satisfied between glucose and lactate concentrations:

(2.60) $$\begin{array}{l} 2C_{M0}+W_{M0}=\frac{2}{\alpha }\text{ for }0<\beta T<\alpha X, \end{array}$$

(2.61) $$\begin{array}{l} 2C_{M0}+W_{M0}=2\text{ for }0<\alpha X<\beta T, \end{array}$$

where the differing constants are due to the “initial” information on the characteristics arising from the actual initial conditions for 0 < β*T* < α*X* (Region 1) and the replenishment boundary conditions for 0 < α*X* < β*T* (Region 2).

## 3. Results

### 3.1. Model Behaviour and Comparison

We now discuss and compare results obtained from our reduced models and the full system. This will allow us to understand when each reduced model is a useful systematic reduction.

The longwave approximation model, Equations (2.42), (2.43), and (2.45)-(2.48), is solved numerically using the Chebfun toolbox in MATLAB. For the sublimit approaximation model, Equations (2.49)-(2.52), we obtain an analytical expression for the glucose concentration, and the lactate concentration is numerically computed from Equation (2.50) subject to Equation (2.51) with a Runge-Kutta method using the in-built ODE solver ode45 in MATLAB. For each numerical approach, we perform convergence tests to ensure the results are independent of mesh size (results not shown).

Computationally, there is a significant difference between the models: on a standard desktop, the full problem is solved in O(180s); the longwave approximation in O(20s); and the sublimit approximation in O(4s). That is, there is a nearly ten-fold speed-up in solving the longwave approximation compared to the full model, and the sublimit is five times quicker to solve than the longwave approximation. As we see later, rapid computation of solutions will allow us to perform parameter sensitivity analyses efficiently.

**Figure 4:**
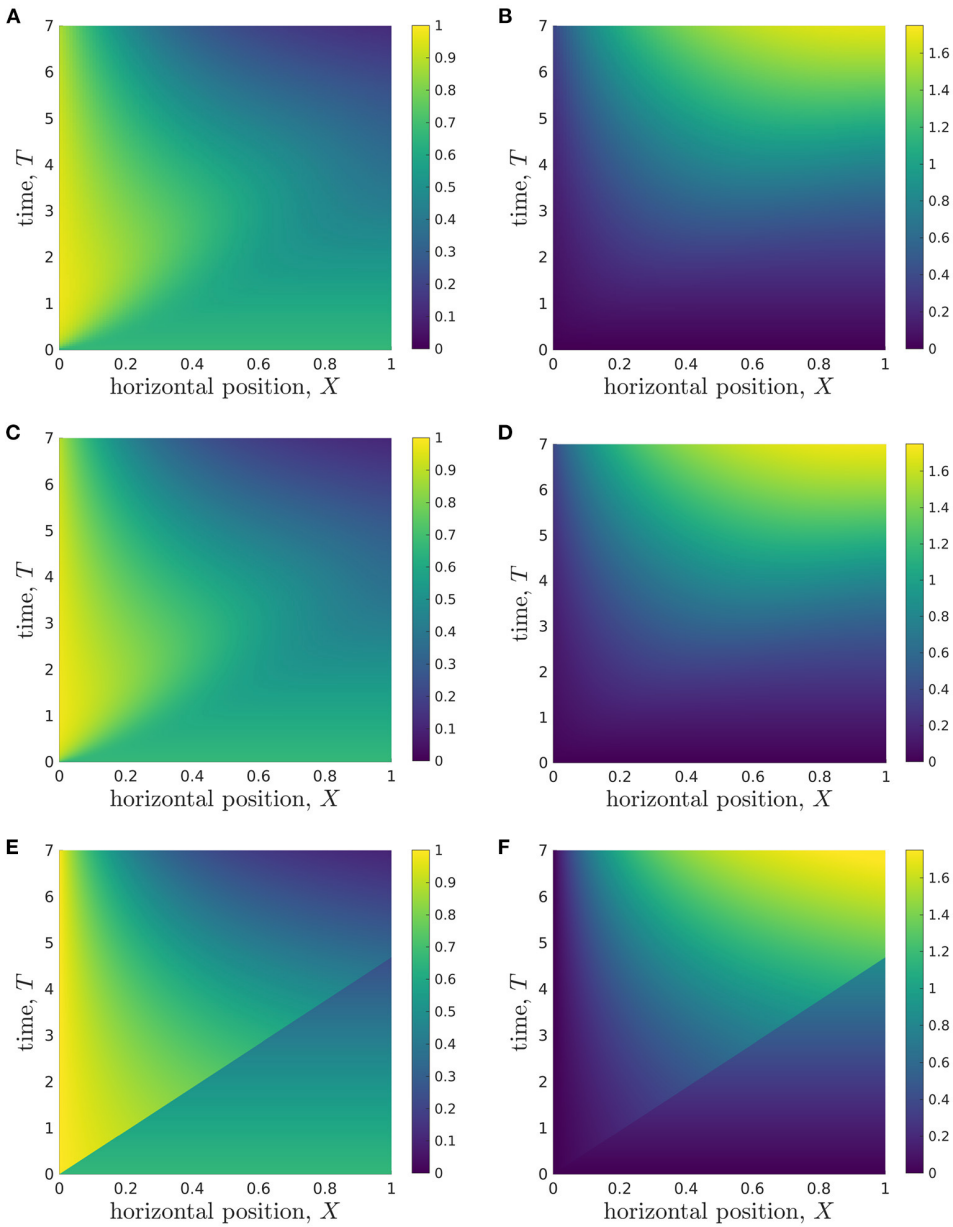
Results showing how the glucose **(A,C,E)** and lactate **(B,D,F)** concentrations change over time during a typical simulation. **(A,B)** Results from *Z*-averaged full model. **(C,D)** Longwave approximation. **(E,F)** Sublimit of longwave approximation, where the upper left and lower right regions are the *replenished* and *unreplenished* regions, respectively. Parameter values: see Table 3.

To present the model solutions over space and time, we average solutions of the full 2D model over *Z*, to facilitate comparison with solutions of the reduced models (Figure 4). We see that the glucose concentration behaviour appears to be split into two approximate regions, divided by a straight line in (*X, T*)-space that goes through the origin and reaches the end of the *X*-domain (*X* = 1) at *T* ≈ 4 (Figure 4A). In the lower-right region, the glucose concentration appears to be approximately constant in space, and to decrease over time. However, in the upper-left region, there is a clear spatial dependence in the glucose concentration, which appears to decrease in *X* until it reaches the lower-right region. The lactate concentration behaviour appears to be split into the same two approximate regions (Figure 4B), though the demarcation is less defined than for glucose. In the lower-right region, the lactate concentration also appears to be approximately constant in space, but now increases over time. In the upper-left region, the lactate concentration appears to approximately increase in *X* until it reaches the lower-right region. To compare these results with the reduced models, we also present solutions for the longwave approximation (Figures 4C,D) and sublimit approximation (Figures 4E,F). We see that the longwave approximation is an excellent approximation of the full system through the entire domain. The sublimit is also a good approximation of the full model except in a small neighbourhood of the dividing characteristic, α*X* = β*T*. The sublimit solution is discontinuous across the dividing characteristic because it neglects horizontal diffusion. Appropriate smoothing could be included in the sublimit by investigating a thin boundary layer in the neighbourhood of this discontinuity in which diffusive effects are once again important. We also note that the dividing characteristic is in approximately the same place as the boundary between regions noted in the full model in Figures 4A,B. We investigate and interpret this observation below.

At this stage, we conclude that when information close to the dividing characteristic is of interest, the longwave approximation should be used instead of the sublimit approximation. If this information is not important, the sublimit approximation should be used since it is faster to solve than the full model and the longwave approximation, and it admits analytic solutions for glucose concentration.

We emphasise that our analytic solutions in the sublimit approximation allow us to understand observations from the full numerical solutions. That is, we can use our analytic solutions from the sublimit model to physically interpret our results and provide insight into the underlying physical system. For example, the dividing characteristic (α*X* = β*T*) in the sublimit model represents the division between information propagated from the initial and the boundary conditions. Physically, this means that the effect of fresh media is only experienced at position *X* at time *T* = α*X*/β. At earlier times, glucose delivery to organoids at position *X* is due to the glucose initially present in the system. This allows us to determine the *metabolite transit time*. That is, the average time taken for metabolite within the *fresh* media to traverse the entire bioreactor

(3.1) $$\begin{array}{l} T^{*}=\frac{\alpha }{\beta }=\frac{1+\frac{H_{H}}{1-H_{H}}}{\mu Ū}\approx 4.7\text{ days}. \end{array}$$

The above estimate is in good agreement with our observations of the full solution—that different model solutions arise in the two regions on either side of the straight line through the origin that reaches *X* = 1 at *T* ≈ 4. Hence, we now interpret this observation physically; the regions are separate according to whether or not they have experienced fresh media. Since the media does not traverse the bioreactor with a constant velocity, the *metabolite transit time* is not the same as the timescale associated with the maximum flow velocity of the system, [*t*] = 25 h. The relevant timescale is, therefore, not the one associated with the experimentally imposed flow rate, but rather the metabolite transit timescale, which is associated with the averaged velocity distribution of metabolite across the bioreactor.

Additionally, the analytic solution of our sublimit approximation provides insight into why the glucose and lactate concentration appear to be spatially-independent in the lower-right regions (Figure 4). In Region 1 (where 0 < β*T* < α*X*), the analytical solutions for metabolite concentrations from the sublimit model are independent of the spatial coordinate. Region 1 is the non-replenished region, *i.e*. it is not replenished from the inlet and subsists on its initial conditions. Given spatially-uniform initial conditions, spatial effects are not seen in the concentration profiles until the wave of replenishment is experienced; this marks the onset of Region 2.

To quantitatively compare the model predictions, we consider the following time-dependent variables: *minimum glucose concentration*, $C_{\min}\left(T\right)=\underset{X}{\min}\left(C\left(X,T\right)\right)$; *maximum lactate concentration*, $W_{\max}\left(T\right)=\underset{X}{\max}\left(W\left(X,T\right)\right)$; *spatial position of maximum lactate concentration*, *X*_max_(*T*), where *W*(*X*_max_, *T*) = *W*_max_(*T*); and the *lactate concentration at outlet*, *W*(*X* = 1, *T*). We emphasise that Equation (2.32) allows us to denote the metabolite concentrations *C*_*M*0_ = *C*_*H*0_ = *C* and *W*_*M*0_ = *W*_*H*0_ = *W* for ease of notation.

**Figure 5:**
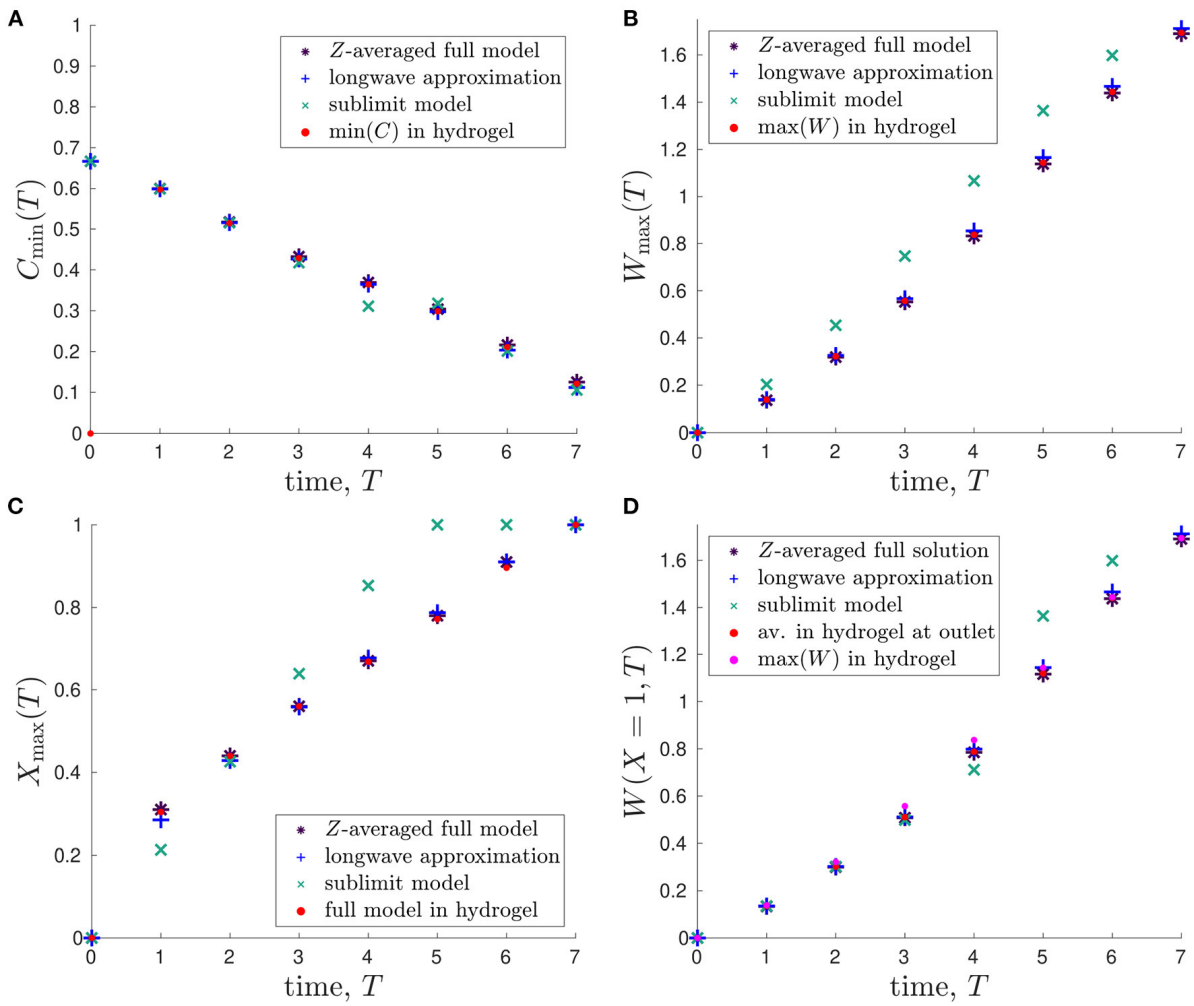
Comparison of outputs from the different mathematical models and their evolution in time: **(A)** minimum glucose concentration, *C*_min_(*T*); **(B)** maximum lactate concentration, *W*_max_(*T*); **(C)** spatial position of maximum lactate concentration, *X*_max_(*T*) s.t. *W*(*X*_max_, *T*) = *W*_max_(*T*); **(D)** lactate concentration at outlet of bioreactor, *W*(*X* = 1, *T*). The red points represent the values predicted in the hydrogel region of the full 2D model. Parameter values: see Table 3.

In Figure 5A, we plot the minimum glucose concentration, *C*_min_(*T*), against time for our two reduced one-dimensional models and the *Z*-averaged full model and compare these values to the predicted minimum glucose concentration in hydrogel, which is found using the full two-dimensional model. We see that the predicted minimum glucose from each model reduction generally agrees well with the minimum glucose within the hydrogel from the full model. The only exceptions to this are around 4-5 days, where the sublimit model disagrees slightly with the other models, and for early times (< 1 day). The first of these is due to the dividing characteristic being important for this metric around 4.7 days, as discussed above. The second is due to our choice of timescale in deriving the reduced model. That is, our reduced models focus over the timescale of days and neglect the initial transient behaviour in the system, as mentioned previously.

Similar plots showing how the maximum lactate concentration, *W*_max_(*T*), changes over time are presented in Figure 5B. Again, the *Z*-averaged full model and the longwave approximation are in good agreement with the predicted value within the hydrogel. Given that there is initially no lactate in the system, this metric avoids the issue with the early time transient behaviour that occurs for the minimum glucose concentration metric. The sublimit approximation systematically overestimates the lactate concentration, though we note that this is preferable to underestimation, given the detrimental effects of high lactate concentrations. The overestimation arises because the sublimit approximation neglects the removal effect of lactate transport through horizontal diffusion over the dividing characteristic.

We compare the position at which the maximum lactate concentration occurs, *X*_max_(*T*), in Figure 5C. We see that *X*_max_ is increasing in time, which is consistent with advection being the dominant transport mechanism over the timescale of days (Table 2), as the lactate produced is advected towards the outlet by the media. As seen in Figures 5A,B, the sublimit approximation agrees less well with the full model than the longwave approximation, which has excellent agreement.

It is infeasible to obtain experimental data for maximum lactate concentrations, which we would need to validate our model. Therefore, we consider the lactate concentration at the media outlet, *W*(*X* = 1, *T*), which is measurable empirically, in Figure 5D. We compare the reduced models to the *Z*-averaged full solution, the average concentration within the hydrogel at the outlet, and the maximum value in the hydrogel (which are all obtained from numerical solutions to the full 2D system). We find that the lactate concentration at the media outlet is very similar to the maximum lactate concentration within the hydrogel and can, therefore, be used as a proxy for it. The sublimit is a good prediction of the outlet and maximum lactate concentrations at 4 days and earlier, but overestimates the maximum concentration within the hydrogel at 5 days and later. This is again due to the dividing characteristic, and its exit from the domain at 4.7 days.

### 3.2. Bioreactor Characterisation

In this section, we start by exploiting our reduced modelling approach to *characterise* the conditions within the bioreactor. We show how the metabolite concentrations depend on the bioreactor operating parameters such as the inlet flow rate and cell seeding density, and the characteristics of the cells, such as the rates of cell proliferation and glucose consumption. Armed with this insight, we then show how the operating parameters can be selected to ensure the biochemical environment within the bioreactor promotes cell growth.

We investigate and quantify the metabolite behaviour by introducing the following time-dependent metrics. We previously defined the *maximum lactate concentration*, *W*_max_(*T*), as

(3.2) $$\begin{array}{l} W_{\max}\left(T\right)=\underset{X}{\max}\left(W\left(X,T\right)\right). \end{array}$$

We now introduce the cumulative *glucose conversion*, *Q*(*T*), as

(3.3) $$\begin{array}{l} Q\left(T\right)=\frac{\text{glucose consumed}}{\text{glucose supplied}}=\frac{\int_{0}^{T}\int_{0}^{1}\gamma C\exp\left(PT\right)\text{ d}X\text{d}T}{\int_{0}^{T}\left(1-H_{H}\right)\mu Ū\text{ d}T}. \end{array}$$

In general, it is desirable to choose operating parameters that ensure high glucose conversion, so the maximum amount of glucose supplied to the bioreactor is utilised by the cells, and resource wastage is minimised. However, high glucose conversion will also cause high lactate levels, and lactate concentrations above a critical tolerance, *W*_tol_, can adversely affect organoid growth. To assess this, we define a point *X* to be *uninhabitable* if *W*(*X, T*) > *W*_tol_. We use the metric *proportion of domain which is uninhabitable*, ***P*_*U*_**(*T*), defined as

(3.4) $$\begin{array}{l} P_{U}\left(T\right)=\int_{0}^{1}H\left[W\left(X,T\right)-W_{\text{tol}}\right]\text{ d}X, \end{array}$$

where *H* is the Heaviside function. In general, it is desirable to choose operating parameters such that ***P*_*U*_** is minimised for the duration of the bioreactor run. In addition to the time-dependent metrics, it is also helpful to quantify the *time at which intolerable lactate levels are first experienced*, which we refer to as the *turn-off time*, and define as

(3.5) $$\begin{array}{l} T_{\text{off}}=\min\left(T\right)\text{ for }T\in \left\{T:W\left(X,T\right)\geq W_{\text{tol}}\right\}. \end{array}$$

In general, it is desirable to choose operating parameters such that *T*_off_ is larger than the duration of the bioreactor run.

There is a trade-off between high glucose conversion and minimising the fraction of the domain which is *uninhabitable*. We show how the mathematical model can be used to identify parameter regimes which strike a balance between promoting glucose conversion and facilitating waste removal in section 3.2.2.

In addition to the metrics we have introduced to assess metabolite distribution, an important cell-specific metric is the *glucose consumption rate per cell*. In our model, the glucose consumption rate per cell is proportional to the glucose concentration and, thus, we can use results such as Figure 4C to understand the spatial variation in glucose consumption rate per cell. We see that cells nearer the inlet have higher rates of glucose uptake than those closer to the media outlet, and this spatial heterogeneity could lead to spatial variation in cell growth within the physical system.

#### 3.2.1. Characterising Model Behaviour for Different Organoid Lines

**Figure 6:**
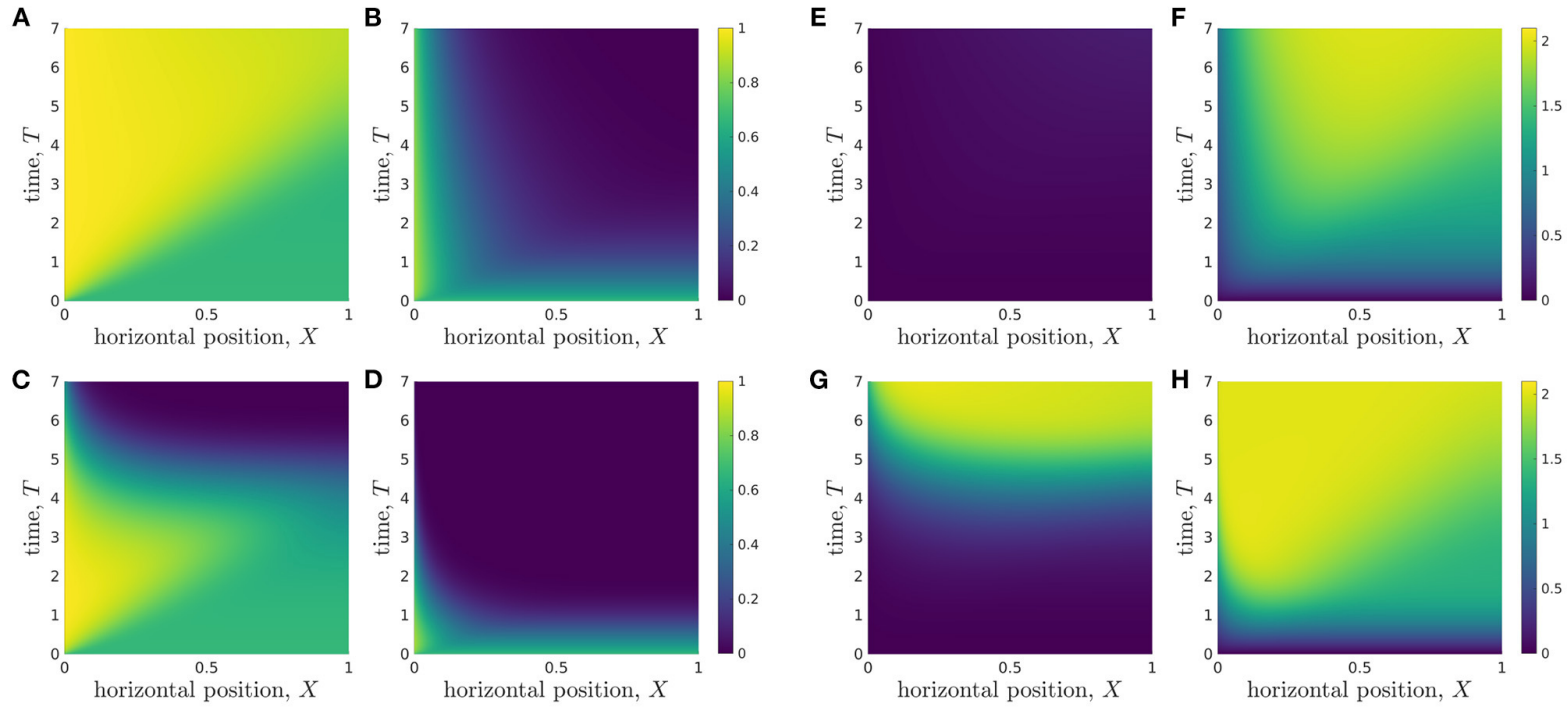
Evolution of glucose **(left grid)** and lactate **(right grid)** concentration profiles over the duration of a typical experiment for different organoid lines under the same operating conditions. The rates of cell proliferation rates and glucose consumption per cell are: **(A,E)** organoid line (i), *p* = 1/6, ρ = 0.027; **(B,F)** organoid line (ii), *p* = 1/6, ρ = 2.7; **(C,G)** organoid line (iii), *p* = 1, ρ = 0.027; **(D,H)** organoid line (iv), *p* = 1, ρ = 2.7. The other parameters used are given in Table 3.

Organoid lines differ in many ways including, but not limited to, proliferation rate, glucose consumption rate, the maximum lactate concentration cells can tolerate without affecting cell properties, and minimum glucose level needed for cellular proliferation. To understand the metabolic environment experienced by different organoid lines within the bioreactor, we perform a discrete parameter sensitivity analysis in which we vary the rates of proliferation, *P*, and glucose consumption per cell, ρ, for the bioreactor operating regime specified in Table 3. We consider organoid lines whose proliferation rates take the values *P* = 1/6 and *P* = 1, which we refer to as low and high proliferation, respectively, and whose glucose consumption rates take values ρ = 0.027 and ρ = 2.7, referred to as low and high consumption, respectively. We consider five different organoid lines: (i) with *P* = 1/6 and ρ = 0.027; (ii) with *P* = 1/6 and ρ = 2.7; (iii) with *P* = 1 and ρ = 0.027; (iv) with *P* = 1 and ρ = 2.7; and the *typical organoid line* considered in Figure 4 (v) with *P* = 1/3 and ρ = 0.27. In Figure 6, we plot the metabolite concentration profiles *C* and *W* for these four organoid lines, (i-iv), expanded under an operating regime which does not otherwise differ. The same results for organoid line (v) are shown in Figures 4C,D.

In Figures 6A,E, we show organoid line (i), cells with low proliferation and low glucose uptake rates. The lactate levels are very low throughout the bioreactor domain and the domain remains within tolerable lactate concentrations for the entire experiment. The glucose concentration in the replenished region is high and remains close to its inlet value, *C* = 1, so the media flow supplies significantly more glucose into the system than is consumed by the cells. The glucose concentration becomes increasingly homogeneous as time evolves, and consequently the rate of glucose consumption per cell becomes more spatially homogeneous across the bioreactor as time evolves.

We consider organoid line (ii), with low proliferation and high glucose uptake rates, in Figures 6B,F. We see that this larger uptake rate means that the lactate concentration quickly increases and the majority of the region becomes intolerable, even for slowly proliferating cells. While cells close to the inlet still have reasonably high glucose and low lactate levels, resulting in the rate of glucose uptake per cell being high at the inlet, this quickly decreases as one moves into the bioreactor.

For rapidly proliferating cells with a low rate of glucose uptake [organoid line (iii)] Figures 6C,G, we see the environment is tolerable until around day 4 of the experiment. At this point, there are approximately 55 times more cells within the hydrogel than at the start of the experiment. This suggests that the selected operating conditions provide tolerable conditions and allow reasonable rate of glucose consumption per cell up to a critical number of cells, but beyond this critical number, the low glucose concentration means the cells have a very low rate of glucose consumption. The lactate concentration is reasonably spatially homogeneous, which suggests that all cells will be subject to a similar metabolic environment and therefore be affected by lactate to a similar degree.

Finally, we consider cells with high proliferation and high uptake [organoid line (iv)], in Figures 6D,H. The glucose concentration within the bioreactor decays very quickly over the course of a day, and it is never replenished sufficiently by the media flow. As such, the glucose consumption per cell is consistently small away from the inlet region. In the same vein, the lactate concentration quickly increases to above the tolerable level over the course of a day. In contrast to the low proliferation organoid line (ii) (Figures 6B,F), the maximum lactate concentration for organoid line (iv) occurs close to the inlet rather than in the middle of the bioreactor. This is because the rapid expansion of cells means that lactate is produced very quickly throughout the bioreactor, and so is maximised in the location where glucose is mainly consumed. This indicates that the media flow is too slow to facilitate significant waste removal for this organoid line. We note that our cell growth model is not dependent on metabolite concentration, so the cell proliferation rate is unaffected when the metabolic environment is harsh. This limitation is most prominent for the high proliferation and high uptake organoid line, where the cells continue to proliferate exponentially in the presence of no glucose and high lactate levels.

**Figure 7:**
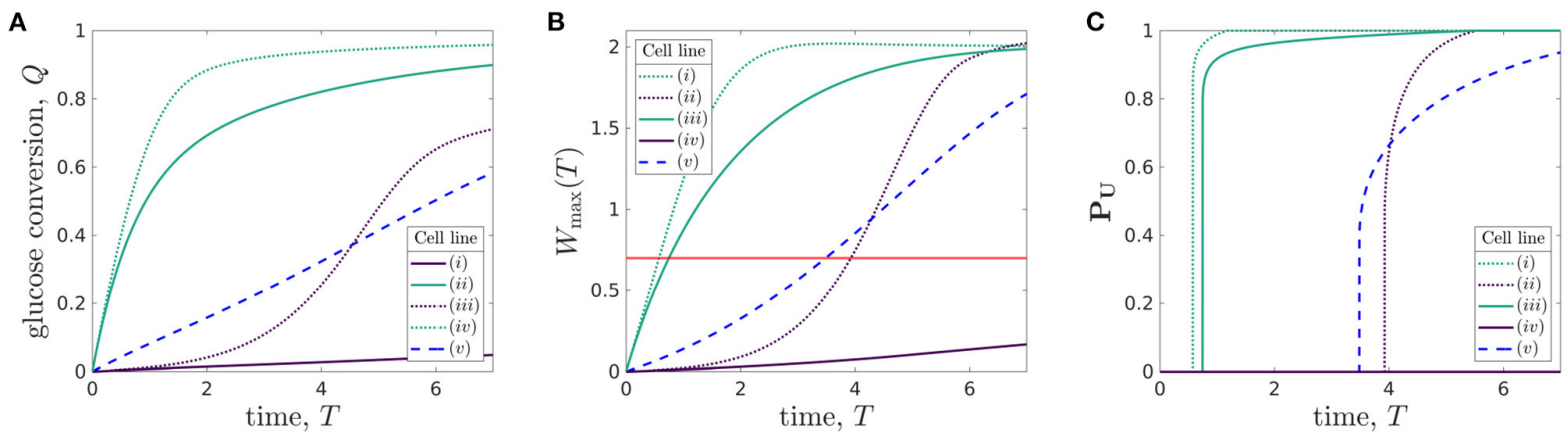
Comparison of **(A)** glucose conversion *Q*, Eqaution (3.3), **(B)** maximum lactate concentration *W*_max_(*T*), Equation (3.2), where the red line represents the maximum tolerated lactate concentration, *W* = *W*_tol_, and **(C)** proportion of domain which is uninhabitable at time *T*, ***P*_*U*_**, Equation (3.4), for different organoid lines cultured within the bioreactor under the same operating conditions. The proliferation rates and rate of glucose consumption per cell for each organoid line are: (i) *p* = 1/6, ρ = 0.027, (ii) *p* = 1/6, ρ = 2.7, (iii) *p* = 1, ρ = 0.027, (iv) *p* = 1, ρ = 2.7, and (v) *p* = 1/3, ρ = 0.27. The other parameters used are given in Table 3. The line styles correspond to rate of cellular proliferation: solid, *P* = 1/6; dashed, *P* = 1/3; and dotted, *P* = 1. The line colours correspond to rate of glucose consumption per cell density: purple, ρ = 0.027; blue, ρ = 2.7; and green, ρ = 2.7.

Using the metrics we introduced above, we now quantify the behaviour of the bioreactor environment during cell culture for each of the five organoid lines. In Figure 7, we plot the total glucose conversion, *Q*(*T*) (Equation 3.3), maximum lactate concentration, *W*_max_(*T*) (Equation 3.2), and proportion of uninhabitable domain, ***P*_*U*_**(*T*) (Equation 3.4) (strongly related to the turn-off time), for each of the five organoid lines.

The glucose conversion generically increases over time, as the cells grow. However, the shape of this increase over time varies significantly between the different organoid lines. While solely considering the standard case [organoid line (v), given by parameters in Table 3] would suggest that the glucose conversion is approximately linear in time, the additional organoid lines show that this behaviour is not universal. Cells with high rates of glucose consumption [organoid lines (ii) and (iv)] have a sharp increase in glucose conversion over the first 2 days before plateauing. For low rates of glucose consumption, the shape of the glucose conversion curve strongly depends on the cell proliferation rate. For low proliferation [organoid line i], the conversion is low throughout and appears linear. However, for high proliferation [organoid line (iii)], the curve has an S-shape. That is, the conversion starts off low, then rapidly increases before plateauing. This rapid increase is linked to the increase in the number of cells in the bioreactor for organoid line (iii), and so we would expect organoid line (i) to exhibit a similar S-shape if the experiment went on for longer.

We show the maximum lactate concentration in Figure 7B, where the red line represents *W* = *W*_tol_, to understand which of these organoid lines are growing in tolerable environments. This graph is qualitatively very similar to that of the glucose conversion, Figure 7A. For the value of *W*_tol_ we use, we see that the maximum lactate concentration reaches the tolerated level within 1 day for high uptake cells [organoid lines (ii) and (iv)]. In comparison, the standard case [organoid line (v)] reaches the maximum tolerated level approximately halfway through the experiment. For the low uptake organoid lines, the proliferation rate again makes a significant difference. For high proliferation [organoid line (iii)], the maximum tolerated level is again reached approximately halfway through the experiment, whereas for low proliferation [cell line (i)] the lactate never reaches harmful levels.

We examine the time at which the lactate concentration equals the tolerated lactate concentration in Figure 7C, a graph showing the time-dependent proportion of the domain which is uninhabitable, ***P*_*U*_**(*T*), for each organoid line. Notably, we see that as soon as some of the domain becomes uninhabitable, the rest of the domain follows over a short timescale. This can be explained through the insight gained from our sublimit approximation. That is, as Region 1 (α*X* > β*T* > 0) has yet to experience replenishment from the inlet, the lactate concentration in this region is approximately spatially homogeneous, and an increase above the tolerable level will quickly be experienced in a large part of the domain. The turn-off time *T*_off_ (Equation 3.5) can also be determined from Figure 7C—it is the first time at which ***P*_*U*_**(*T*) is non-zero. We see that the high glucose consumption organoid lines [(ii) and (iv)] have much smaller turn-off times than the other organoid lines. The lactate concentration for organoid line (i) does not reach *W*_tol_ during the experiment, so the turn-off time is larger than the run time of the experiment.

There is a trade-off between promoting: (1) high glucose conversion, to ensure resources are not wasted; (2) high glucose consumption rate per cell, to ensure cells absorb sufficient glucose to proliferate; and (3) increasing the turn-off time, to ensure the lactate concentrations within the bioreactor remain tolerable everywhere throughout the experiment. Our model framework allows for efficient quantification of all these metrics. By determining how these metrics vary with bioreactor operating parameters, we can then identify operating conditions that enhance cell growth. We illustrate this in the next section.

#### 3.2.2. Determining Operating Conditions for a Given Organoid Line

In this subsection, we focus on the standard organoid line (*v*), with proliferation rate and glucose consumption rate given in Table 3. This is the organoid line with a “medium" rate of glucose consumption per cell, and a doubling time of 3 days. The current operating conditions lead to lactate concentrations above the tolerated level for half of the experimental run time, suggesting that these operating conditions are sub-optimal.

We now determine how the metrics depend on the inlet flow rate for this organoid line, and show how this leads to the identification of flow rates that enhance cell growth. We focus on flow rate as this is an experimental parameter that is easily varied. We investigate flow rates over two order of magnitudes, [*u*] ∈ [1 × 10^−7^, 1 × 10^−5^]m s^−1^, all within the range of the peristaltic pump used in the CXP1 protocol.

**Figure 8:**
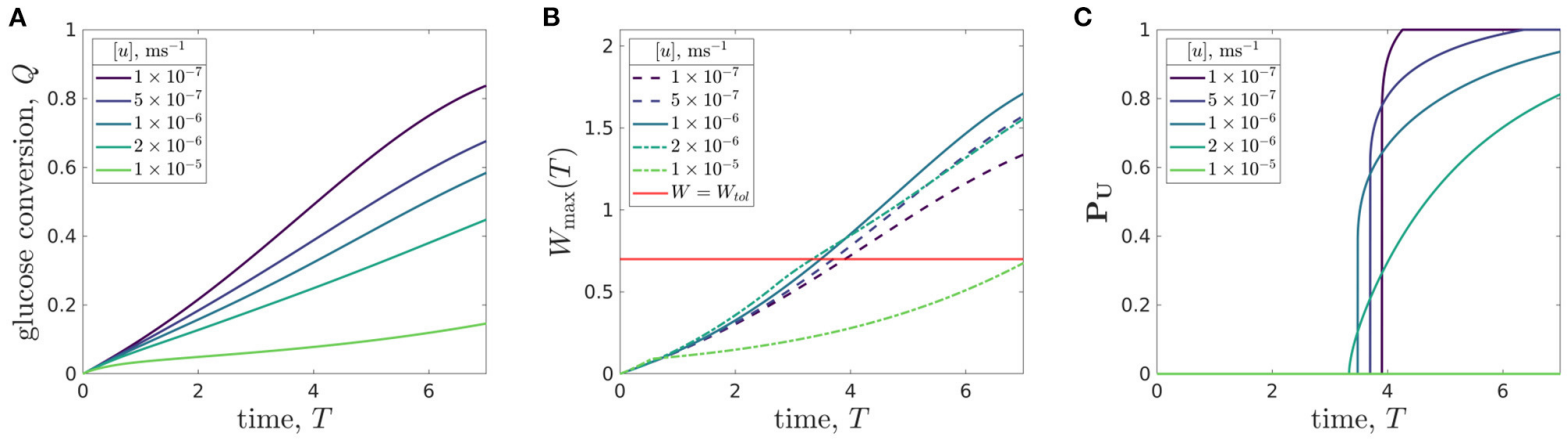
Results for a specific organoid line within the CXP1 bioreactor showing the evolution of: **(A)** glucose conversion *Q*, Equation (3.3), **(B)** maximum lactate concentration *W*_max_(*T*), Equation (3.2), where the red line respresents the maximum tolerated lactate concentration, *W* = *W*_tol_, and **(C)** proportion of domain which is uninhabitable at time *T*, ***P*_*U*_**, Equation (3.4), against time for five different flow rates. For [*u*] = 10^−5^m s^−1^, the value of ***P*_*U*_** is zero. The peak flow velocities [*u*] ∈ {10^−7^, 5 × 10^−7^, 10^−6^, 2 × 10^−6^, 10^−5^}m s^−1^ used correspond to the dimensionless flow velocity parameter μ ∈ {0.096, 0.48, 0.96, 1.92, 9.6}, respectively. Remaining parameter values: see Table 3.

In Figure 8, we show how the metrics vary with inlet flow rate. To illustrate the dependence of the metrics on flow rate, we first present time-dependent results for five different flow rates. The glucose conversion monotonically increases in time (Figure 8A), due to the increasing number of cells causing an increased glucose consumption. The effect of increasing flow rate is to decrease the glucose conversion. This is because stronger flows correspond to feeding more glucose into the system over a given time period as well as the media spending less time within the bioreactor, so there is less time for the glucose to be consumed by the cells. However, we also note that the conversion is relatively insensitive to flow rate: increasing the flow by two orders of magnitude only decreases the conversion by a factor of around six.

While the time-dependent maximum lactate concentration within the domain monotonically increases for a given flow rate, the effect of varying the flow rate is non-monotonic (Figure 8B). For a given run time of the experiment, there is a flow rate that maximises the maximal lactate concentration. We emphasise that this flow rate will depend on the experimental run time. The reason for there being a flow rate which maximises the maximal lactate concentration (the “worst” flow rate, in some sense) is due to two competing factors. Firstly, the rate of glucose consumption per cell, and therefore the rate of lactate production, increases with increasing flow rate. Secondly, for slower flow rates the media is not able to advect sufficient quantities of lactate out of the bioreactor to maintain a tolerable lactate level. These two factors combine to produce a worst possible flow rate for a given experimental run time. We also note that up until approximately one day (*T* = 1), the maximum lactate concentration is the same for all the flow rates considered. This reflects the fact that there is a lag in the production of lactate, and that the lactate production is initially set by the initial conditions rather than the operating regime of the bioreactor.

In Figure 8C, we plot the proportion of the domain which is uninhabitable against time, for the five different flow rates considered. In general, a lower flow rate corresponds to a sharper increase in the uninhabitable proportion once initially triggered. This is because more of the domain is in the non-replenished Region 1 for lower flow rates, and the metabolite concentrations are approximately spatially independent in Region 1, for reasons discussed above. In addition, we note that a large enough flow rate can ensure that none of the domain becomes uninhabitable for the duration of the experimental run, as we see for a flow rate of 1 × 10^−5^m s^−1^. However, we also note that increasing the flow rate can have an unwanted effect on the turn-off time. From Figure 8C, we see that increasing the flow rate slightly decreases the turn-off time, up to a point. As noted above, for large enough flow rates the system never exhibits intolerable lactate concentrations.

**Figure 9:**
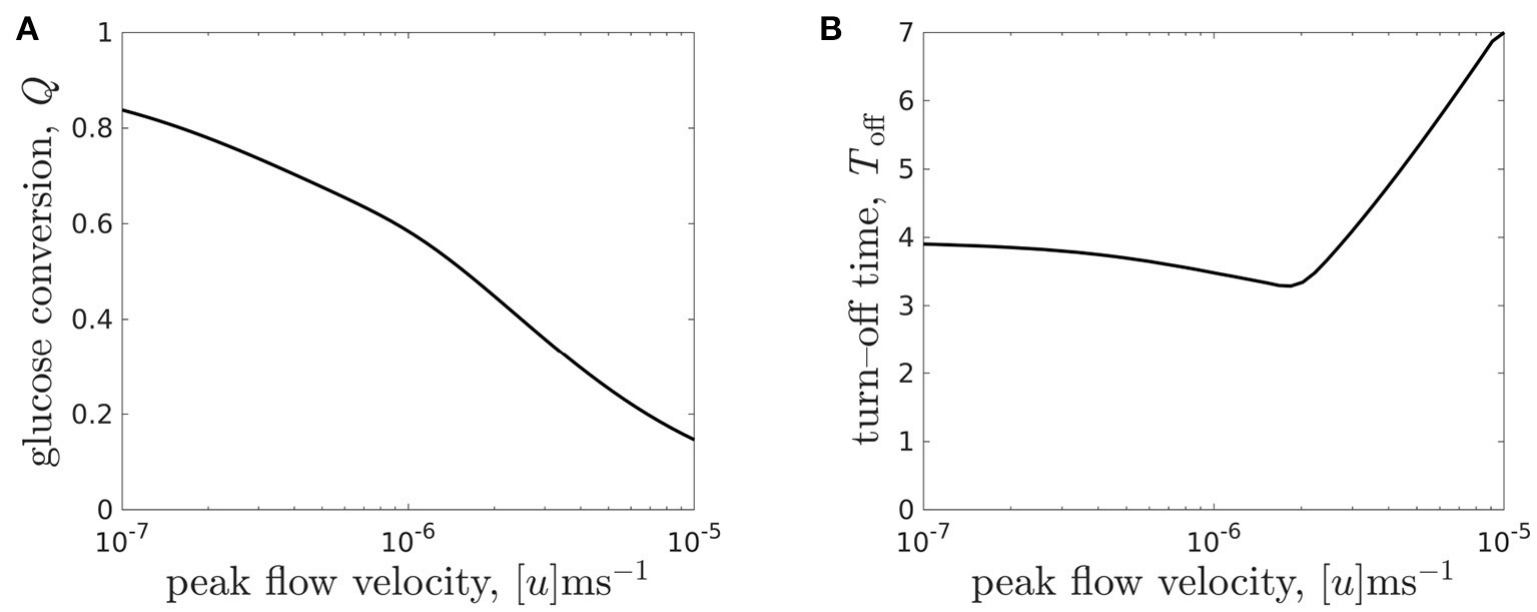
**(A)** Glucose conversion *Q*, Equation (3.3), at time *T* = 7 and **(B)** turn-off time *T*_off_ (the time when intolerable lactate levels first experienced) for the CXP1 bioreactor varying with flow rate, for a given organoid line. Peak flow velocities [*u*] ∈ [10^−7^, 10^−5^]m s^−1^ correspond to dimensionless flow rate, μ, in the range μ ∈ [0.096, 9.6] and the other parameter values are given in Table 3.

We now consider a more finely refined investigation of the effect of flow rate of the system metrics. In Figure 9, we consider the effect of flow rate both on the glucose conversion at day 7 (Figure 9A) and on the turn-off time (Figure 9B).

We see that the relationship between glucose conversion at 7 days and media flow velocity is monotonically decreasing, and the rate of decrease is larger for flows faster than [*u*] = 10^−6^m s^−1^ (Figure 9A). However, as noted above, the turn-off time is not monotonic in the flow rate (see also Figure 8C). We see that there is a minimal turn-off time when the flow is approximately 2 × 10^−6^m s^−1^. This is the *worst* possible flow rate from the point of view of ensuring the domain remains tolerable for as long as possible. For flow rates below this, the bioreactor is *transport-limited*, either by insufficient glucose delivery to cells or by insufficient waste removal from the bioreactor. For flow rates above this, the turn-off time is *proliferation-limited*, where the rate at which the cell population is growing sets the timescale at which lactate is produced.

An advantage of our mathematical modelling framework is that we have been able to easily explore a wide range of parameter values, in this case the flow rate, and explore the nonlinear effects of varying experimental parameters. For example, an experimentalist may start with a slow flow rate of 10^−7^m s^−1^ and conduct a set of experiments over which they increased the flow. Over an order of magnitude increase in flow, they would see no improvement in turn-off time, and therefore might be discouraged from increasing the flow any further. In such a scenario, they would miss finding the flow rate values required for turn-off times greater than 4 days.

The “optimal” operating conditions for the bioreactor will determine glucose and lactate concentrations which (1) yield a specified value for glucose conversion; (2) maintain a glucose consumption rate per cell which is sufficient for cellular proliferation; and (3) predict a turn-off time which is greater than the run time of the experiment. The specific values and relative importance of each of these requirements will depend on the user. Our model reduction facilitates rapid calculation of each metric. Hence, our work could be combined with an optimisation algorithm, with user-specified cost functions, to produce an efficient framework that can identify the bioreactor operating conditions that optimise for growth of organoids.

## 4. Discussion

We have presented an unsteady, two-dimensional model of metabolite transport that predicts metabolite concentrations within the CXP1 bioreactor system. We used an asymptotic analysis to systematically derive two reduced models which exploit the extreme spatial and temporal parameter ratios in the system. Our model predicts the spatiotemporal distribution of the metabolic environment within the bioreactor, information which is challenging to obtain experimentally. Both reduced models are one-dimensional in space; the *longwave approximation* comprises two coupled reaction-advection-diffusion equations, whereas the *sublimit approximation* comprises two coupled reaction-advection equations. Our systematic analysis allows us to relate parameters in the reduced models to geometric and operating parameters of the CXP1 system, such as the ratio between the depth of the hydrogel and media layers, and the fluid flux over the hydrogel. We have shown that both reduced models provide good approximations of the full model for most physically relevant parameter regimes. The longwave approximation is an excellent representation throughout the entire domain, whereas the sublimit approximation is a good representation everywhere apart from one specific line in space-time that we are able to calculate.

Although the above may appear to suggest that the sublimit approximation is not useful, it does have additional benefits over the longwave approximation. A notable benefit is that it admits analytic solutions in the entire domain. Interpreting these analytic results, and understanding why they are discontinuous across the specific line in space-time, provides insight into the underlying physical system. We find that the specific line in space-time is a dividing characteristic in the (hyperbolic) sublimit approximation we derive. We are able to infer that this line divides the domain into two regions, depending on whether or not the effect of replenishment from the inlet has been experienced.

The flow of media through the bioreactor has the dual function of delivering nutrients to, and removing waste from, the growing organoids. As such, the inlet flow rate needs to be chosen carefully. The systematic reduction we have performed yields models that are easier to solve numerically than the full model. More importantly, they provide insight into the behaviour of the full model, particularly the dominant transport mechanisms. This systematic reduction has enabled us to efficiently characterise the experimental parameter space for given cell characteristics. One key outcome from this analysis is our prediction of a “worst-case” flow rate that minimises the turn-off time (the time when intolerable lactate concentrations first occur), Equation (3.5). Our model reduction has allowed us to understand why this minimum arises: for higher flow rates, the lactate is washed away more quickly (the bioreactor is in a proliferation-limited regime), for lower flow rates the lactate is produced more slowly since glucose is not delivered quickly enough (the bioreactor is in a transport-limited regime).

To understand how outcomes change as the control parameters are varied, we introduced the following time-dependent metrics which characterise bioreactor performance:

- ***Glucose conversion*** is the ratio between the total amounts of consumed and supplied glucose. It is desirable to minimise the amount of resources, *e.g*. glucose, required for bioreactor operation, which corresponds to maximising glucose consumption.
- ***Maximum lactate concentration*** within the bioreactor represents the worst metabolic environment experienced by the cells. High lactate concentrations have a detrimental effect on cells (Romero-Garcia et al., 2016), and therefore an ideal bioreactor operating regime would have low maximum lactate concentrations.
- ***Proportion of uninhabitable domain*** is the fraction of the domain where the lactate concentrations exceeds the maximum tolerated level for the specific organoid line. An operating regime is improved if the proportion of the domain which is uninhabitable decreases, and an “ideal” operating regime would maintain lactate levels below the maximum tolerable level for the entire experiment.
- ***Turn-off time*** is the time at which lactate concentration first reaches levels which are intolerable for the cells. To optimise operating conditions, the turn-off time should be increased. Ideally, the turn-off time should exceed the run time of the experiment.

Different bioreactor operating conditions will yield different values of these metrics. The relative importance of each metric will depend on the particular organoid line being investigated and the specific user requirements. Our work provides a framework for efficiently determining desirable bioreactor operating conditions for given cell properties.

In this study, we performed a systematic model reduction to study metabolite transport within the CXP1 bioreactor, whose geometry differs significantly from other bioreactors, such as hollow fibre or perfusion bioreactors. An important insight gained from our model reduction is the identification of the transport mechanisms that are dominant on our timescale of interest. We performed model reductions in two ways: (1) we exploited the slender geometry of the system, to obtain the *longwave approximation*; and then (2) we exploited the separation of timescales of the physical processes in play, to derive the *sublimit approximation*. By systematically reducing our original model [Equations (2.16)-(2.19) and (2.22)-(2.29)], we have simplified a two-dimensional parabolic PDE system first to a one-dimensional parabolic PDE system (the *longwave approximation*), and then to a one-dimensional hyperbolic PDE system (the *sublimit approximation*). A significant advantage of this approach is the analytical tractability of the *sublimit approximation*. As a result, we can construct explicit expressions for the metabolite concentrations across the entire bioreactor that reveal both the spatiotemporal-dependence and the dependence on the control parameters, *e.g*. flow rate, of the metabolite concentrations in the bioreactor. We have shown that the reduced models serve as excellent approximations of the full system and are much easier to solve numerically. We have also identified the small region of space-time where the assumptions required for the validity of sublimit model break down.

There are a number of interesting possible extensions to this work. For example, the optimal operating conditions are likely to change during the course of organoid growth. Future modelling work could predict how, and when, operating conditions should change to account for this growth. While we have considered steady flows, it would be straightforward to extend our framework to examine more complex flow behaviours, such as oscillating flows, or three-dimensional effects. The potential use of unsteady flows will be of particular interest when minimisation of spatial variation in metabolite concentrations across the bioreactor is important, as we have seen that steady flows with little spatial variation in metabolite concentration also have very low conversion (see Figures 6A,E, 7A). The ability to change the mathematical flow model when predicting the metabolite concentrations is particularly useful because it can be done in advance of engineering the prototype bioreactors needed to test the system experimentally.

In this work, we considered a spatially constant cell density, with growth rates independent of the local biochemical environment. Future modelling work will represent individual organoids as small, localised regions within the hydrogel where glucose consumption and lactate production occur, and regulate organoid growth. We will use a mathematical homogenisation approach (see *e.g*. Sanz-Herrera et al., 2008; Shipley et al., 2009; Dalwadi et al., 2018; Dalwadi and King, 2020) to systematically average the behaviour over the microscale to obtain a macroscale governing equation for the hydrogel layer with effective *glucose consumption, lactate production*, and *organoid growth* terms. This in turn will increase our understanding of the relationship between the bioreactor operating parameters and the mean and variation in organoid size, ultimately facilitating optimisation of the bioreactor operating conditions to minimise organoid size variation.

The mathematical modelling approach developed in this paper provides a framework for establishing how organoid viability can be improved by varying bioreactor operating conditions. The framework has the flexibility to consider different organoid lines, via characterisation of their proliferation and nutrient consumption rates and their tolerance to the presence of waste metabolite. Our work has the potential to improve the quality and reproducibility of bioreactor-expanded organoid output. We intend our theoretical framework to be used to scale-up the production of viable organoids, contributing to overall organoid technology development, and enabling organoids to be exploited as a powerful tool for accelerating drug discovery and testing.

## Data Availability Statement

The datasets generated for this study can be found in the GitHub repository https://github.com/meredithellis/A_reduced-order_model_for_organoid_expansion.

## Author Contributions

All authors designed the research and wrote the paper. MAE performed the research.

## Conflict of Interest

MJE is a co-founder of Cellesce. The remaining authors declare that the research was conducted in the absence of any commercial or financial relationships that could be construed as a potential conflict of interest.
