# Supplementary material for: A Systematically Reduced Mathematical Model for Organoid Expansion
Source: Front Bioeng Biotechnol. 2021 Jun 10;9:670186. doi: 10.3389/fbioe.2021.670186 (PMC8222789; doi:10.3389/fbioe.2021.670186)
Supplement: Supplementary file 1 [file Data_Sheet_1.pdf]

Supplementary Material

1 DERIVATION OF ANALYTIC SOLUTION TO THE SUBLIMIT APPROXIMATION

We consider the sublimit approximation discussed in Section 2.3.2,

$$\alpha\frac{\partial C}{\partial T}+\beta\frac{\partial C}{\partial X}=-\gamma C\exp(PT), \tag{1.1}$$
$$\alpha\frac{\partial W}{\partial T}+\beta\frac{\partial W}{\partial X}=2\gamma C\exp(PT), \tag{1.2}$$

with boundary and initial conditions

$$C=1, \quad W=0 \quad \text{at } X=0, \tag{1.3}$$
$$C=\frac{1}{\alpha}, \quad W=0 \quad \text{at } T=0. \tag{1.4}$$

We can make progress analytically via the method of characteristics.

1.1 Solution for glucose concentration

We begin by considering the glucose concentration, since Eq. (1.1) is decoupled from Eq. (1.2), and introduce our coordinates in characteristic space, (S,τ), where we define

$$S=\alpha X-\beta T, \tag{1.5}$$

where S is a constant along each characteristic curve and τ parameterises the characteristics. Each characteristic curve for Eq. (1.1) will satisfy:

$$\frac{\mathrm{d}T}{\mathrm{d}\tau}=\alpha, \tag{1.6}$$
$$\frac{\mathrm{d}X}{\mathrm{d}\tau}=\beta, \tag{1.7}$$
$$\frac{\mathrm{d}C}{\mathrm{d}\tau}=-\gamma Ce^{PT}, \tag{1.8}$$

with our initial data, $\Gamma(S)=(T(S),X(S),C(S))$ at $\tau=0$,

$$\Gamma(S)=\begin{cases}\left(0,\ \frac{S}{\alpha},\ \frac{1}{\alpha}\right) & \text{for } S>0, \quad (1.9)\\ \left(-\frac{S}{\beta},\ 0,\ 1\right) & \text{for } S<0. \quad (1.10)\end{cases}$$

Solving Eqs. (1.6) and (1.7) subject to the initial data gives the coordinate transformations,

$$\text{For } S>0: \begin{cases}T=\alpha\tau,\\ X=\beta\tau+\frac{S}{\alpha},\end{cases} \quad \text{and for } S<0: \begin{cases}T=\alpha\tau-\frac{S}{\beta},\\ X=\beta\tau\end{cases} \tag{1.11}$$

Region 1, $S > 0$:

The equation for C, Eq. (1.8), becomes

$$\frac{\mathrm{d}C}{\mathrm{d}\tau} = -\gamma C e^{P\alpha\tau}, \tag{1.12}$$

which we integrate to obtain

$$C = D(S)\exp\left(-\frac{\gamma}{P\alpha}e^{P\alpha\tau}\right), \tag{1.13}$$

where $D(S)$ is an arbitrary function of S to be determined from the initial condition, $C(S,\tau=0) = 1/\alpha$. We obtain

$$C(S,\tau) = \frac{1}{\alpha}\exp\left(\frac{\gamma}{P\alpha}\right)\exp\left(-\frac{\gamma}{P\alpha}e^{P\alpha\tau}\right) \quad \text{for } S > 0, \tag{1.14}$$

which we can rewrite in Cartesian coordinates as:

$$C(X,T) = \frac{1}{\alpha}\exp\left(\frac{\gamma}{\alpha P}\left(1 - e^{PT}\right)\right), \text{ which holds for } \alpha X - \beta T > 0. \tag{1.15}$$

Region 2, $S < 0$:

The equation for C, Eq. (1.8), becomes

$$\frac{\mathrm{d}C}{\mathrm{d}\tau} = -\gamma C e^{P\left(\alpha\tau - \frac{S}{\beta}\right)}, \tag{1.16}$$

which we integrate to obtain

$$C = E(S)\exp\left(-\frac{\gamma}{P\alpha}e^{P\left(\alpha\tau - \frac{S}{\beta}\right)}\right), \tag{1.17}$$

where $E(S)$ is an arbitrary function of S to be determined from the boundary condition, $C(S,\tau=0) = 1$. We obtain

$$C(S,\tau) = \exp\left(\frac{\gamma}{P\alpha}e^{-P\frac{S}{\beta}}\right)\exp\left(-\frac{\gamma}{P\alpha}e^{P\left(\alpha\tau - \frac{S}{\beta}\right)}\right) \quad \text{for } S < 0, \tag{1.18}$$

which we can rewrite in Cartesian coordinates as:

$$C(X,T) = \exp\left(\frac{\gamma}{\alpha P}\left(e^{-P\left(\frac{\alpha}{\beta}X - T\right)} - e^{PT}\right)\right), \text{ which holds for } \alpha X - \beta T < 0. \tag{1.19}$$

1.2 Solution for lactate concentration

In a similar manner, we can use the method of characteristics to make analytic progress for the lactate concentration, W. We introduce our characteristic coordinates S and τ again, and the governing equation, Eq. (1.2), satisfies:

$$\frac{\mathrm{d}T}{\mathrm{d}\tau} = \alpha, \quad \frac{\mathrm{d}X}{\mathrm{d}\tau} = \beta, \quad \frac{\mathrm{d}W}{\mathrm{d}\tau} = 2\gamma C e^{PT}, \tag{1.20}$$

with initial data, $\tilde{\Gamma}(S) = (T(S), X(S), W(S))$ at $\tau = 0$:

$$\tilde{\Gamma}(S) = \begin{cases} \left(0,\ \dfrac{S}{\alpha},\ 0\right) & \text{for } \ S > 0, \quad (1.21) \\ \left(-\dfrac{S}{\beta},\ 0,\ 0\right) & \text{for } \ S < 0. \quad (1.22) \end{cases}$$

We have the same coordinate transformation for Regions 1 and 2 as for C, Eq. (1.11). Hence, we can write the governing equation for W as an explicit integral of known functions:

$$W(S,\tau) = \int_0^{\tau} 2\gamma C\left(T(S,\tau), X(S,\tau)\right) e^{PT(S,\tau)}\, \mathrm{d}\tau, \ \text{subject to } W = 0 \text{ at } \tau = 0, \tag{1.23}$$

where S is treated as a constant and C is defined in Eqs. (1.14) and (1.18).

Region 1, $S > 0$:

For Region 1 ($S > 0$), Eq. (1.20) becomes,

$$\frac{\mathrm{d}W}{\mathrm{d}\tau} = 2\gamma\frac{1}{\alpha}\exp\left(\frac{\gamma}{P\alpha}\right)\exp\left(-\frac{\gamma}{P\alpha}e^{P\alpha\tau}\right)e^{P\alpha\tau}, \tag{1.24}$$

We integrate to solve this explicitly, to find

$$W = -\frac{2}{\alpha}e^{\frac{\gamma}{P\alpha}}\exp\left(-\frac{\gamma}{P\alpha}e^{P\alpha\tau}\right) + F(S), \tag{1.25}$$

where the arbitrary function $F(S)$ is determined by the boundary condition $W = 0$ at $\tau = 0$ to give

$$W(S,\tau) = \frac{2}{\alpha}\left(1 - e^{\frac{\gamma}{P\alpha}}\exp\left(-\frac{\gamma}{P\alpha}e^{P\alpha\tau}\right)\right), \tag{1.26}$$

which we can rewrite in Cartesian coordinates as:

$$W(X,T) = \frac{2}{\alpha}\left(1 - \exp\left(\frac{\gamma}{P\alpha}\left(1 - e^{PT}\right)\right)\right). \tag{1.27}$$

Region 2, $S < 0$:

For Region 2 ($S < 0$), Eq. (1.20) becomes,

$$\frac{\mathrm{d}W}{\mathrm{d}\tau} = 2\gamma\exp\left(\frac{\gamma}{P\alpha}e^{-P\frac{S}{\beta}}\right)\exp\left(-\frac{\gamma}{P\alpha}e^{P\left(\alpha\tau-\frac{S}{\beta}\right)}\right)e^{P\left(\alpha\tau-\frac{S}{\beta}\right)}. \tag{1.28}$$

Similarly, we integrate this to find

$$W = -2\exp\left(\frac{\gamma}{P\alpha}e^{-\frac{PS}{\beta}}\right)\exp\left(-\frac{\gamma}{P\alpha}e^{P\left(\alpha\tau-\frac{S}{\beta}\right)}\right) + G(S), \tag{1.29}$$

where the arbitrary function $G(S)$ is determined by the boundary condition $W = 0$ at $\tau = 0$ to give:

$$W(S,\tau) = 2\left(1 - \exp\left(\frac{\gamma}{P\alpha}\left(e^{-P\frac{S}{\beta}} - e^{P\left(\alpha\tau - \frac{S}{\beta}\right)}\right)\right)\right), \tag{1.30}$$

which we can rewrite in Cartesian coordinates as:

$$W(X,T) = 2\left(1 - \exp\left(\frac{\gamma}{P\alpha}\left(e^{-P\left(\frac{\alpha}{\beta}X - T\right)} - e^{PT}\right)\right)\right). \tag{1.31}$$
